# *Lachnospiraceae-bacterium* alleviates ischemia-reperfusion injury in steatotic donor liver by inhibiting ferroptosis via the Foxo3-Alox15 signaling pathway

**DOI:** 10.1080/19490976.2025.2460543

**Published:** 2025-01-30

**Authors:** Shenghe Deng, Huan Cao, Tongxi Li, Xueling Wang, Junpeng Meng, Teng Zeng, Di Zhang, Shuhua Zhang, Guoliang Wang, Ran Liu, Tianhao Zou, Mao Cai, Ren Lang, Di Lu, Jinyang Gu

**Affiliations:** a Center for Liver Transplantation, Union Hospital, Tongji Medical College, Huazhong University of Science and Technology, Wuhan, Hubei, China; b Department of General Surgery, The Second Hospital of Shanxi Medical University, Taiyuan, China; c Department of Hepatobiliary Surgery, Beijing Chao-Yang Hospital Affiliated to Capital Medical University, Beijing, China; d Key Laboratory of Integrated Oncology and Intelligent Medicine of Zhejiang Province, Department of Hepatobiliary and Pancreatic Surgery, Affiliated Hangzhou First People’s Hospital, Zhejiang University School of Medicine, Hangzhou, Zhejiang Province, China; e Institute of Organ Transplantation, Zhejiang University, Hangzhou, Zhejiang Province, China; f Key Laboratory of Organ Transplantation, Ministry of Education; NHC Key Laboratory of Organ Transplantation; g Key Laboratory of Organ Transplantation, Chinese Academy of Medical Sciences, Wuhan, Hubei, China

**Keywords:** *Lachnospiraceae-bacterium*, steatotic donor livers, ferroptosis, liver transplantation, Alox15

## Abstract

Ischemia-reperfusion injury (IRI) is a major obstacle in liver transplantation, especially with steatotic donor livers. Dysbiosis of the gut microbiota has been implicated in modulating IRI, and *Lachnospiraceae* plays a pivotal role in regulating host inflammatory and immune responses, but its specific role in liver transplantation IRI remains unclear. This study explores whether *Lachnospiraceae* can mitigate IRI and its underlying mechanisms. We found *Lachnospiraceae-bacterium* (*Lachn*.) abundance was significantly reduced in rats with liver cirrhosis. *Lachn*.-treated rats exhibited improved intestinal permeability, reduced IRI severity in both normal and steatotic donor livers, and decreased levels of neutrophil and macrophage infiltration, and inflammatory cytokines. Multi-omics analysis revealed elevated pyruvate levels in transplanted livers after *Lachn*. treatment, alongside reduced Alox15 and Foxo3 expression. Mechanistically, *Lachn*.-derived pyruvate inhibited Alox15 expression and reduced ferroptosis in normal and steatotic donor livers. Furthermore, reduced nuclear translocation of Foxo3 further suppressed Alox15 expression, alleviating IRI, especially in steatotic donor livers. Clinical samples confirmed reduced donor livers IRI in cirrhotic recipients with high *Lachn*. abundance after liver transplantation. In conclusion, *Lachn*. alleviates IRI in steatotic donor liver transplantation by inhibiting ferroptosis via the Foxo3-Alox15 axis, providing a potential therapeutic strategy to modulate gut microbiota to alleviate IRI following liver transplantation.

## GRAPHICAL ABSTRACT

**Figure:**
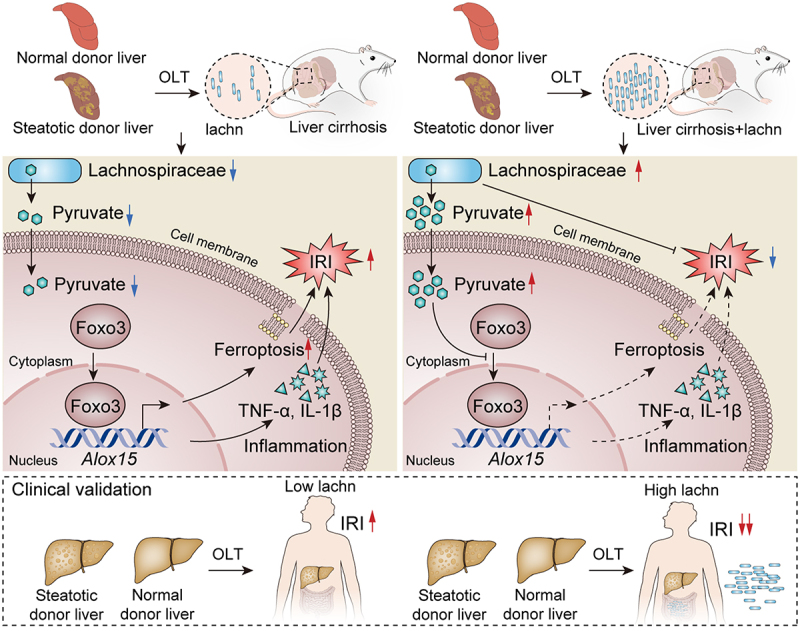

## Introduction

With the rising incidence of liver disease, liver transplantation (LT) has become the primary intervention for treating end-stage liver disease.^1^ However, the ongoing shortage of donor organs has led to an increased use of marginal donor livers, such as from older adult donors, donations following circulatory death, livers infected with hepatitis viruses, and steatotic donor livers.^2,3^ Among these, steatotic donor livers are of particular concern because the growing prevalence of metabolic dysfunction-associated steatohepatitis driven by shifts in dietary patterns underscores the need to optimize their use.^4,5^ Unfortunately, owing to structural and functional abnormalities, steatotic livers are particularly vulnerable to ischemia-reperfusion injury (IRI), which increases the risk of perioperative complications during transplantation.^6,7^ The effective control of IRI in transplanted livers, particularly in steatotic donor livers, is critical for reducing acute liver injury, preventing transplant failure, mitigating complications, prolonging graft survival, and could expand the pool of usable donor livers and help address the shortage of suitable organs.^8,9^ Consequently, addressing IRI, particularly in steatotic donor livers, represents a significant clinical challenge that requires urgent solutions.

Numerous cellular and molecular mechanisms are involved in IRI. Various cell types, such as hepatic sinusoidal endothelial cells, hepatocytes, Kupffer cells, neutrophils, and platelets, contribute to these processes via interconnected molecular pathways, which include the activation of Toll-like receptor signaling, alterations in microRNA expression, production of reactive oxygen species, regulation of autophagy, and activation of hypoxia-inducible factors.^10,11^ Recent studies have found that ferroptosis is a newly recognized form of regulated cell death and have confirmed its significant role in driving hepatic and renal IRI. Ferroptosis refers to iron- and lipid peroxidation-dependent cell death, characterized by increased iron accumulation, impaired lipid repair systems, and lipid peroxidation, which activate inflammatory responses and ultimately lead to membrane damage and cell death.^12,13^ In LT, the donor liver undergoes both warm and cold ischemic phases, the longer the ischemic time, the more severe the IRI after reperfusion, particularly for steatotic donor livers. However, it remains unclear whether ferroptosis is closely associated with the extent of IRI in donor livers during LT. Currently, only a few studies have suggested that the ferroptosis mechanism may play an important role in LT, with inhibition of ferroptosis alleviating donor liver IRI.^14,15^ However, due to the complexity of the donor liver tissue microenvironment, further research is needed to elucidate the mechanisms by which ferroptosis contributes to IRI, especially in steatotic donor livers, and to determine whether targeting ferroptosis could offer a therapeutic strategy for mitigating IRI in liver transplantation.

In recent years, the role of the gut microbiota in host health and disease has attracted significant attention. Studies have shown that dysbiosis of the gut microbiota is closely linked to the onset of various diseases, including inflammatory bowel disease, cancer, obesity, cardiovascular diseases, and diabetes.^16–18^ Additionally, interventions targeting the gut microbiota alleviate IRI in the liver, heart and kidneys by reducing apoptosis, ferroptosis, lowering inflammatory responses, and decreasing immune cell recruitment.^19–21^ Studies have found that oleanolic acid, produced by gut microbiota, can inhibit ferroptosis and thus prevent fatty liver IRI.^22^ Additionally, reducing the gut microbiota metabolite trimethylamine N-oxide has been shown to decrease ferroptosis in myocardial cells, alleviating myocardial cell IRI.^23^ However, despite ongoing research, the specific effects and mechanisms by which different microbiota alleviate organ transplant-related IRI remain unclear. *Lachnospiraceae*, a core component of the gut microbiota that is known for its beneficial properties, can ferment various substrates and produce numerous metabolites.^24^ It reportedly exhibits anti-inflammatory effects, induce immune responses, and helps maintain homeostasis. Moreover, *Lachnospiraceae* can indirectly influence peripheral organs through the activation of hormonal and neural pathways, presenting significant therapeutic potential.^25–27^ Research has shown that supplementing *Lachnospiraceae* and its products can alleviate intestinal inflammation and rheumatoid arthritis in mice by inhibiting ferroptosis.^28,29^ In liver injury studies, *Lachnospiraceae* has also been shown to suppress ferroptosis, thereby reducing acute liver injury in mice.^21^ Nonetheless, whether *Lachnospiraceae* can inhibit ferroptosis, inflammatory response and reduce donor livers especially in steatotic donor livers IRI in clinical LT remains to be determined and warrants further investigation.

In this study, we elucidated the mechanisms by which *Lachnospiraceae* mitigates IRI following LT. Our findings revealed that *Lachnospiraceae-bacterium (Lachn.)* can alleviate IRI in both normal and steatotic donor liver transplants. Specifically, pyruvate derived from *Lachn*. suppresses the expression of hepatic forkhead box O3 (FOXO3) and arachidonate 15-lipoxygenase (ALOX15), thereby reducing liver inflammation, susceptibility to ferroptosis, and the infiltration of inflammatory immune cells, ultimately alleviating IRI. Thus, preoperative modulation of *Lachn*. abundance is a potentially promising therapeutic strategy for mitigating IRI in LT.

## Materials and methods

### Animal models

#### Metabolic dysfunction-associated steatotic liver disease (MASLD) model

To establish the MASLD rat model, male rats (Sprague-Dawley), starting at 14 weeks of age, were fed a high-fat methionine-choline-deficient diet (HFD) for two weeks. Control animals received a normal standard diet (ND). All rats were housed in a specific pathogen-free environment, with a maximum of three rats per cage, and had ad libitum access to food and water throughout the experiments. Criteria for successful MASLD model: Each donor liver must be confirmed as having severe fatty liver through both H&E staining and Oil Red O staining. The rats were euthanized by an overdose of isoflurane followed by cervical dislocation prior to liver and serum sample collection.

#### Liver fibrosis model

The liver fibrosis rat model was induced using subcutaneous injections of CCl_4_ in male rats. Each rat received a single injection dose of 2.5 ml/kg (20% concentration in olive oil), administered twice weekly for eight weeks, starting at eight weeks of age. Control animals were injected with saline. Criteria for a successful liver fibrosis model: H&E staining indicates significant hepatocyte damage, chronic inflammatory cell infiltration in adjacent liver tissues, and localized or extensive nodular lesions encapsulated by fibrous septa; Masson staining reveals extensive blue collagen fiber deposition, extending outward from the periportal area, with thick fibrous strands forming pseudo-lobules. The rats were euthanized by an overdose of isoflurane followed by cervical dislocation prior to sample collection.

#### Rat orthotopic liver transplantation (OLT) model

The OLT procedure was performed on rats (normal and CCl_4_ treated rats) at 16 weeks of age. Buprenorphine was administered pre-operatively to both donor and recipient rats. Rats were anesthetized using 50 mg/kg of pentobarbital sodium, and the surgical site was sterilized with a 70% ethanol and betadine solution. The portal vein was skeletonized, and the livers were perfused with 4°C UW solution and excised. After 18 hours of cold storage, the donor livers were implanted into healthy recipient rats. Following reperfusion for six hours, blood samples were collected from the inferior vena cava. The livers were then perfused with normal saline from the portal vein and excised. Each group consisted of at least five rats. To avoid potential variations caused by surgical techniques, all liver transplantation procedures were performed by HC. All animal studies received approval from the Animal Ethics Committee of Tongji Medical College, Huazhong University of Science and Technology (No. 2023–3996).

*Additional methods are provided in the Supplemental methods.*

## Results

### *Increased intestinal permeability and decreased* Lachn. *abundance in liver cirrhosis rats*

Colonic and fecal tissues were collected from 12 pairs of normal and liver cirrhosis (LC) rats, and we observed increased intestinal permeability in LC rats compared to controls, as confirmed by western blotting, electron microscopy (Figure S1A-C), and FITC-dextran (FD4) assays (Figure S1D). Metagenomic analyses of fecal samples revealed significant microbial differences between the two groups at the phylum, genus, and species levels (Figure S1E-H). Notably, fluorescence in situ hybridization (FISH) further validated a reduced abundance of the core intestinal bacterium *Lachnospiraceae* (Lachn.) and an increase in *Prevotella* species in LC rats (Figure S1I, Figure S2A).

Further analysis revealed a negative correlation between *Lachn*. abundance and liver alanine transaminase (ALT) and aspartate transaminase (AST) levels, whereas *Prevotella-sp*. abundance was positively correlated (Figure S2B). Functional analysis of fecal microbiota revealed enrichment in metabolic pathways (Figure S2C). Untargeted metabolomics of feces identified significant differences in metabolites between groups, particularly those linked to metabolic pathways (Figure S2D, E). Notably, LC rat feces exhibited lower levels of short-chain fatty acids (SCFAs) and organic acids (valeric, pyruvic, and succinic acids), which are known to protect against intestinal permeability and are positively correlated with *Lachn*. abundance (Figure S2H). These findings suggest that changes in *Lachn*. abundance may influence intestinal permeability and merit further exploration, especially in the context of IRI following LT in LC recipients.

### Lachnospiraceae *improves intestinal permeability and alleviates steatotic donor liver IRI*

**Figure 1.**
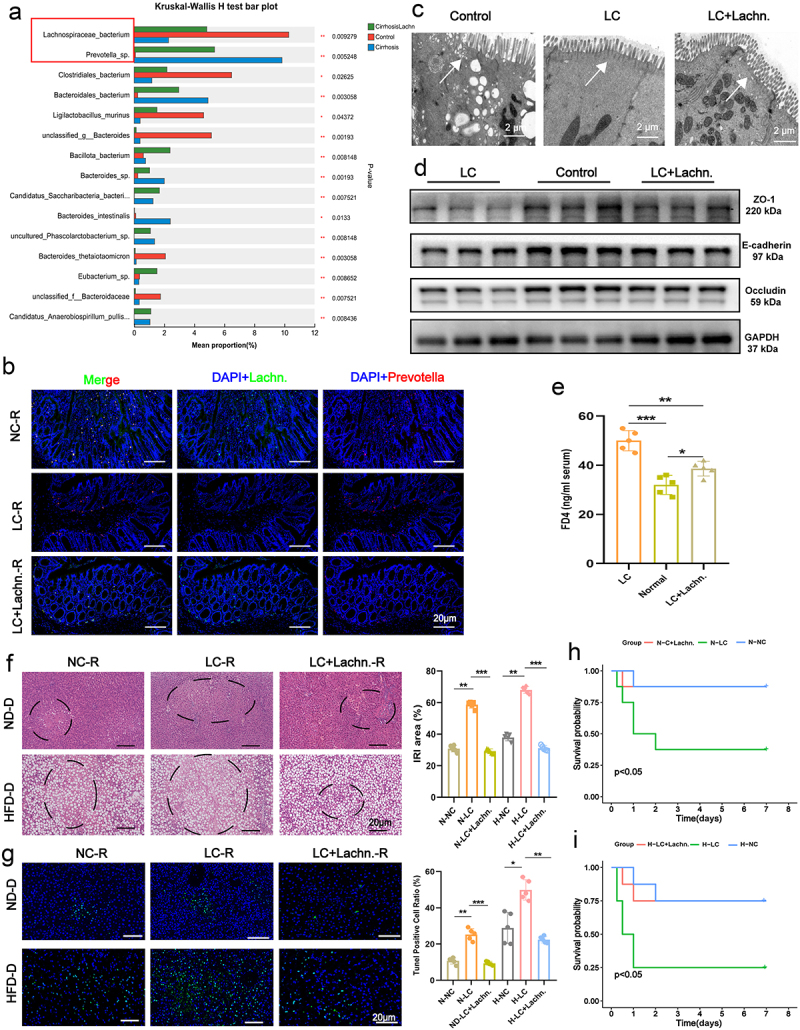
*Lachn*. improves intestinal permeability and alleviates steatotic donor liver IRI. (a) heatmap showing changes in the fecal microbiota of liver cirrhosis (LC) rats after treatment with *Lachn*. (LC+Lachn.). (b) fluorescence *in situ* hybridization (fish) experiments confirmed changes in *Lachn*. and *Prevotella-sp*. in the colon of normal (NC-R), LC-R, and LC+Lachn. rats (LC+Lachn.-R) (scale bar = 20 μm). (c – e) Electron microscopy (c), western blot (d), and FD4 assays (e) assessing intestinal permeability in normal rats, LC-R, and LC+Lachn.-R (scale bar = 2 μm). (f) Hematoxylin and eosin (H&E) staining of liver iri in normal, LC-R, and LC+Lachn.-R following transplantation with normal diet donor liver (ND-D) and high-fat diet donor liver (HFD-D) (scale bar = 20 μm). (g) terminal deoxynucleotidyl transferase dUTP nick end labeling (tunel) assay for liver iri in normal, LC-R, and LC+Lachn.-R post-transplantation with ND-D and HFD-D livers (scale bar = 20 μm). (H,I) survival analysis 1 week post-transplantation with ND-D and HFD-D livers in normal, LC-R, and LC+Lachn.-R. Statistical significance was determined using unpaired Student’s t-test. *p < 0.05, **p < 0.01, ***p < 0.001.

*Lachn*. have been reported to play a crucial role in modulating host inflammatory responses and immune status.^24–27^ To explore whether *Lachn*. can mitigate IRI post-LT, we administered *Lachn*. via gavage to LC rats (LC+Lachn.-R). Fecal samples metagenomic and untargeted metabolomic analysis revealed significant changes in microbial composition after *Lachn*. intervention, including increased *Lachn*. abundance and reduced *Prevotella-sp* species (Figures 1a, Figure S3A-E). Furthermore, gut microbiota functions were enriched in metabolic pathways (Figure S3F). FISH analysis confirmed the increased abundance of *Lachn*. and decreased *Prevotella* in colon tissues of LC+Lachn.-R rats (Figure 1b). Electron microscopy, western blotting, tissue and cellular polymerase chain reaction (PCR), and FITC-dextran (FD4) assays confirmed that intestinal epithelial cell alignment was tighter and permeability significantly improved in LC+Lachn.-R rats compared to LC rats (LC-R) (Figures 1c-e, Figure S3G, H).

To evaluate IRI in LT, both normal and steatotic donor livers (normal diet-donor and high fat diet-donor, ND-D and HFD-D) were used. Hematoxylin and eosin (H&E) staining, TUNEL assay, and ELISA showed that *Lachn*. intervention significantly reduced ALT and AST levels, alleviating donor liver IRI, particularly in steatotic donor livers (Figures 1f, g, Figure S3I). Additionally, inflammatory cytokines were markedly reduced in transplanted livers (Figure S4A, B). Western blotting and PCR confirmed decreased levels of tumor necrosis factor (TNF)-α and interleukin (IL)-6. Furthermore, significant downregulation of C-X-C motif chemokine ligand 2 (CXCL2), high mobility group box 1 (HMGB1), and monocyte chemoattractant protein-1 (MCP1) expression was observed in both the liver tissues and portal vein serum. Liver tissues immunofluorescence staining showed significant reduction in neutrophil (CD11b) and macrophage (CD68) infiltration, particularly in steatotic donor livers (Figures S4C, D). Finally, survival analysis showed that LC+Lachn.-R rats receiving either normal or steatotic donor livers had significantly higher survival rates 1 week post-LT compared to LC-R rats, with a more pronounced effect in transplants involving steatotic donor livers (Figure 1h, i). Therefore, these findings indicate that *Lachn*. enhances intestinal permeability, reduces inflammation in transplanted livers, and alleviates IRI in both normal and steatotic donor livers.

### *Validation of the mitigating effect of* Lachn. *in steatotic donor LT IRI in antibiotic-treated liver cirrhosis (LC+ABX) rats*

**Figure 2.**
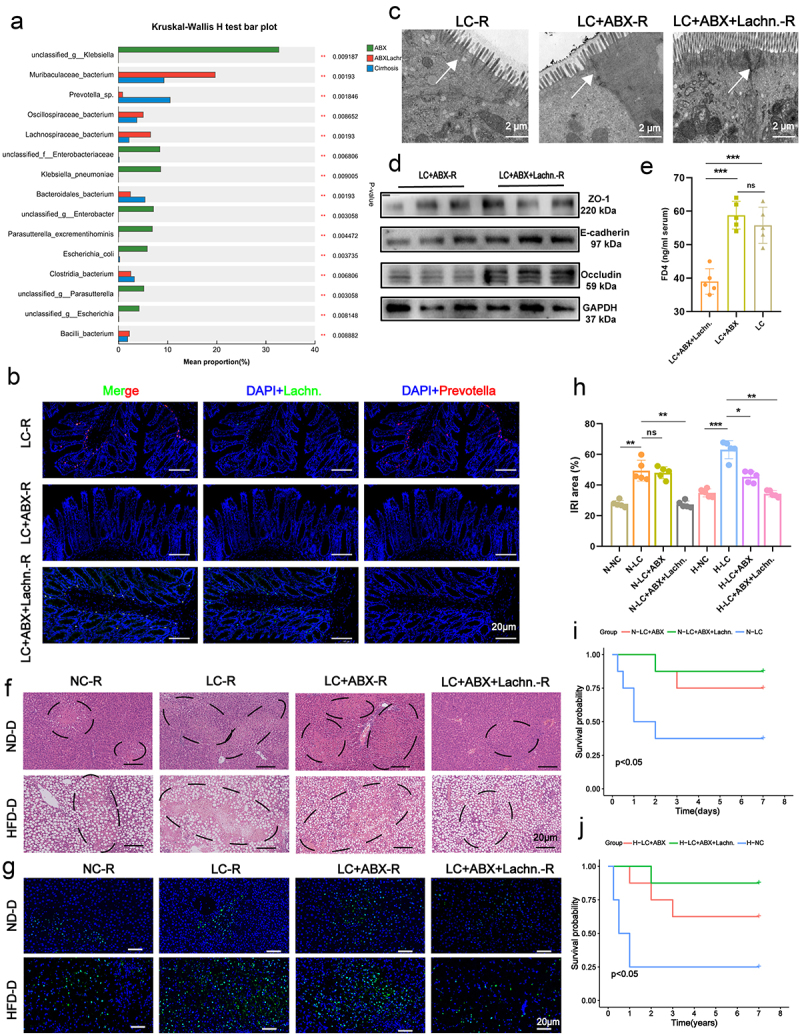
Validation of the mitigating effect of *Lachn*. in steatotic donor LT IRI in antibiotic-treated liver cirrhosis (LC+ABX) rats. (a) heatmap illustrating changes in fecal intestinal microbiota following *Lachn*. intervention in abx-treated rats with liver cirrhosis (LC+ABX+Lachn.). (b) fluorescence *in situ* hybridization (fish) analysis confirmed the presence and variation of *Lachn*. and *Prevotella-sp*. in the colons of rats with LC, abx-treated rats with LC (LC+ABX), and LC+ABX+Lachn (scale bar = 20 μm). (c – e) Electron microscopy (c), Western blotting (d), and fluorescein isothiocyanate (FITC)-dextran assays (e) performed to assess intestinal permeability in LC-R, LC+ABX-R, and LC+ABX+Lachn.-R (scale bar = 2 μm). (f – h) Hematoxylin and eosin (H&E) and terminal deoxynucleotidyl transferase dUTP nick end labeling (tunel) assays to evaluate liver iri following transplantation of normal diet donor livers (ND-D) and high-fat diet steatotic donor livers (HFD-D) in LC-R, LC+ABX-R, and LC+ABX+Lachn.-R (scale bar = 20 μm). (i,j) one-week survival analysis after transplantation of ND-D and HFD-D livers in LC-R, LC+ABX-R, and LC+ABX+Lachn.-R. Statistical significance was determined using unpaired Student’s t-test. *p < 0.05, **p < 0.01, ***p < 0.001, ns: not significant.

To further explore the role of *Lachn*. in mitigating IRI in LT, we established an LC+ABX rat model and administered *Lachn*. intervention. Similar to the findings in LC+Lachn.-R rats, *Lachn*. treatment in LC-ABX rats (LC-ABX+Lachn.-R) altered gut microbiota composition, with increased *Lachn*. abundance and decreased *Prevotella* levels (Figure 2a, Figure S5A-D). Untargeted metabolomics revealed changes in microbial metabolic functions. Intestinal tissue analysis via FISH, electron microscopy, western blotting, FD4, and PCR showed that *Lachn*. intervention improved intestinal epithelial integrity and significantly enhanced gut permeability (Figure 2b-e, Figure S5 F).

Regarding IRI in transplanted livers, H&E, TUNEL, and ELISA assays demonstrated *Lachn*. substantial mitigation of IRI, with a pronounced protective effect on steatotic donor livers (Figure 2f-h, Figure S5G, H). Inflammatory marker analysis in both liver tissues and portal vein serum revealed that *Lachn*. significantly reduced TNF-α, IL-6, CXCL2, HMGB1, and MCP-1 expression (Figure S6A-C). Additionally, Additionally, neutrophil and macrophage infiltration into transplanted livers was notably decreased following *Lachn*. treatment (Figures S6D, E). Survival analysis of LC-ABX rats subjected to LT revealed that *Lachn*.-treated rats, particularly those receiving steatotic donor livers, had significantly improved survival rates (Figures 2i,j). These results confirm that *Lachn*. can improve gut permeability and significantly attenuate IRI in transplanted livers.

### *Pyruvate acid from* Lachn. *enhances intestinal permeability and mitigates steatotic donor liver IRI*

**Figure 3.**
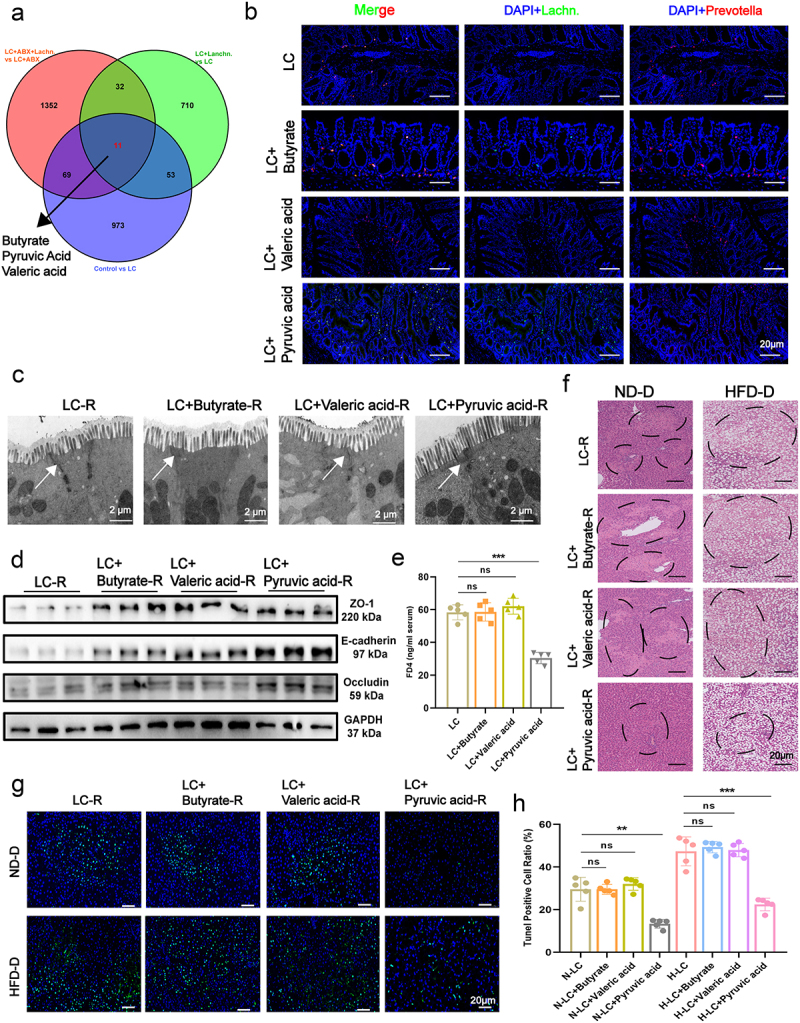
Pyruvate acid from *Lachn*. enhances intestinal permeability and mitigates steatotic donor liver IRI. (a) venn diagram of differential metabolic products post-intervention with *Lachn*. in rats with liver cirrhosis (LC) and abx-treated rats with LC (LC+ABX). (b) fluorescence *in situ* hybridization (fish) analysis to assess changes in *Lachn*. and *Prevotella-sp*. in the colon of LC rats after intervention with pyruvic, valeric, and butyrate acids (scale bar = 20 μm). (c – e) Electron microscopy (c), western blot (d), and fluorescein isothiocyanate (FITC)-dextran assays (e) to evaluate changes in intestinal permeability in LC rats following intervention with pyruvic, valeric, and butyrate acids (scale bar = 2 μm). (f – h) Hematoxylin and eosin (H&E) and terminal deoxynucleotidyl transferase dUTP nick end labeling (tunel) assays to determine the degree of liver iri and semi-quantitative analyses post-transplantation of ND-D and HFD-D livers in LC rats treated with pyruvic, valeric, and butyrate acids (scale bar = 20 μm). Statistical significance was determined using unpaired Student’s t-test. **p < 0.01, ***p < 0.001, ns: not significant.

To identify the metabolites from *Lachn*. that alleviate IRI post-LT, we performed untargeted metabolomic analysis of fecal samples from normal (NC-R), LC-R, LC+Lachn.-R, LC-ABX-R, and LC-ABX+Lachn.-R rats. Following *Lachn*. intervention, increased *Lachn*. abundance was accompanied by elevated levels of butyrate, pyruvate, and valeric acid (Figure 3a, Figure S7A). Given the potential roles of these metabolites in intestinal barrier repair and anti-inflammatory responses, we treated LC rats with butyrate, pyruvate, and valeric acid to identify the active metabolite in *Lachn*.. FISH, electron microscopy, western blotting, FD4, and PCR assays revealed that pyruvic acid, but not butyrate or valeric acid, significantly restored epithelial tight junction integrity, improved intestinal permeability, and enhanced *Lachn*. abundance (Figure 3b-e, Figure S7B).

In LT models using both normal and steatotic donor livers, pyruvic acid intervention significantly reduced the post-transplantation serum ALT and AST levels (Figure S7C). Histological analysis with H&E staining and TUNEL assays confirmed a substantial reduction in IRI, particularly in steatotic donor livers (Figure 3f-h, Figure S7D). Additionally, pyruvic acid also significantly reduced liver and serum levels of inflammatory markers, including TNF-α, IL-6, CXCL2, HMGB1, and MCP-1 (Figure S7E, Figure S8A-C). Furthermore, neutrophil and macrophage infiltration into the transplanted liver was significantly reduced following pyruvic acid treatment (Figure S8D). These results demonstrate that pyruvate from *Lachn*. improves gut permeability, reduces inflammation, and alleviates IRI in both normal and steatotic donor livers.

### *Validation of pyruvate acid from* Lachn. *in enhancing intestinal permeability and mitigated steatotic donor liver IRI*

**Figure 4.**
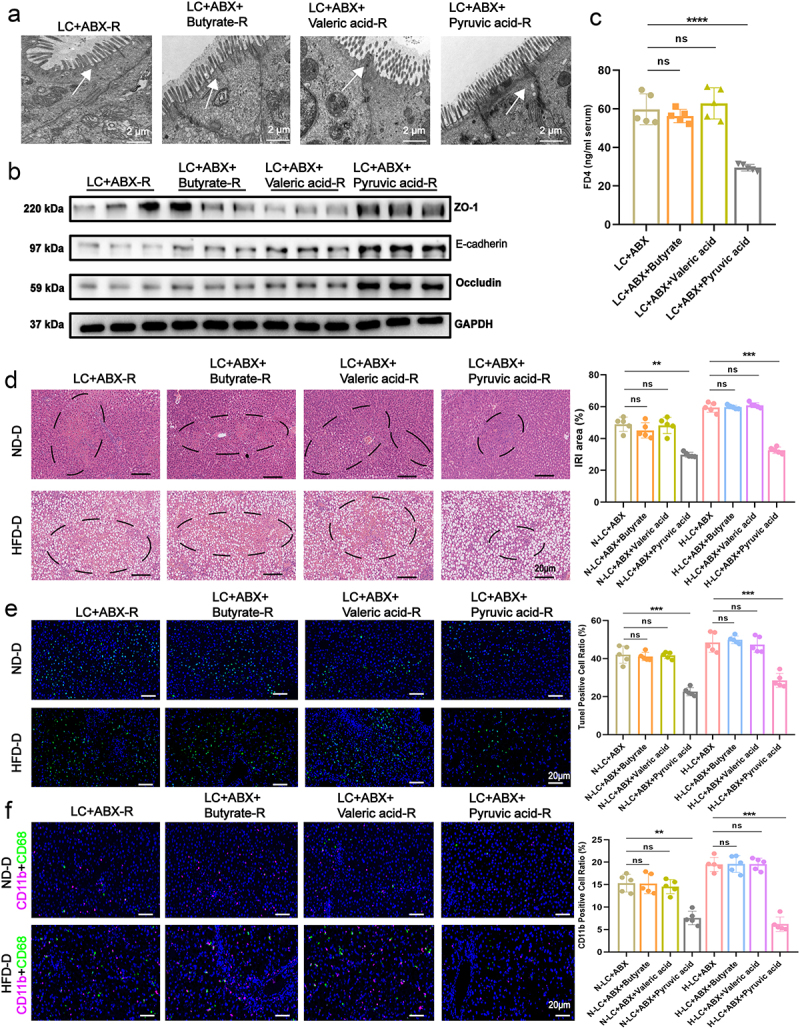
Validation of pyruvate acid from *Lachn*. in enhancing intestinal permeability and mitigated steatotic donor liver IRI. (a – c) Electron microscopy (a), western blot (b), and fluorescein isothiocyanate (FITC)-dextran assays (c) evaluating changes in intestinal permeability in ABX-treated liver cirrhosis rats (LC+ABX) following intervention with pyruvic, valeric, and butyrate acids (scale bar = 2 μm). (d,e) Hematoxylin and eosin (H&E) and terminal deoxynucleotidyl transferase dUTP nick end labeling (tunel) assays to assess the level of liver iri and semi-quantitative analyses post-transplantation of ND-D and HFD-D liver in LC+ABX rats with pyruvic, valeric, and butyrate acids intervention (scale bar = 20 μm). (f) immunofluorescence analysis of neutrophil and macrophage infiltration in the transplant liver of LC+ABX rats after intervention with pyruvic, valeric, and butyrate acids, following transplantation of ND-D and HFD-D livers (scale bar = 20 μm). Statistical significance was determined using unpaired Student’s t-test. **p < 0.01, ***p < 0.001, ****p < 0.0001, ns: no significant.

To further confirm that pyruvate acid derived from *Lachn*. improves intestinal permeability and mitigates IRI during LT, we administered butyrate, pyruvate, and valerate to LC-ABX rats. Intestinal permeability revealed that pyruvate acid significantly tightened epithelial cell junctions (Figure 4a-c, Figure S9A-C). In LT models, pyruvate treatment led to marked reductions in ALT and AST levels, along with decreased IRI severity, compared to butyrate and valerate interventions. Furthermore, pyruvate also significantly lowered the expression of inflammatory markers, including TNF-α, IL-6, CXCL2, HMGB1, and MCP-1, in both the transplanted liver and serum at the gene and protein levels (Figure 4d, e, Figure S9 D, E, Figure S10A-D). Additionally, pyruvate treatment significantly reduced neutrophil and macrophage infiltration in transplanted livers, particularly in steatotic donor livers (Figures 4e, Figures S10E). These findings suggest that pyruvate acid from *Lachn*. improves intestinal permeability and substantially alleviates IRI, with more pronounced effects observed during steatotic donor LT.

### Pyruvate acid mitigates ferroptosis in transplanted liver IRI by inhibiting ALOX15 expression

**Figure 5.**
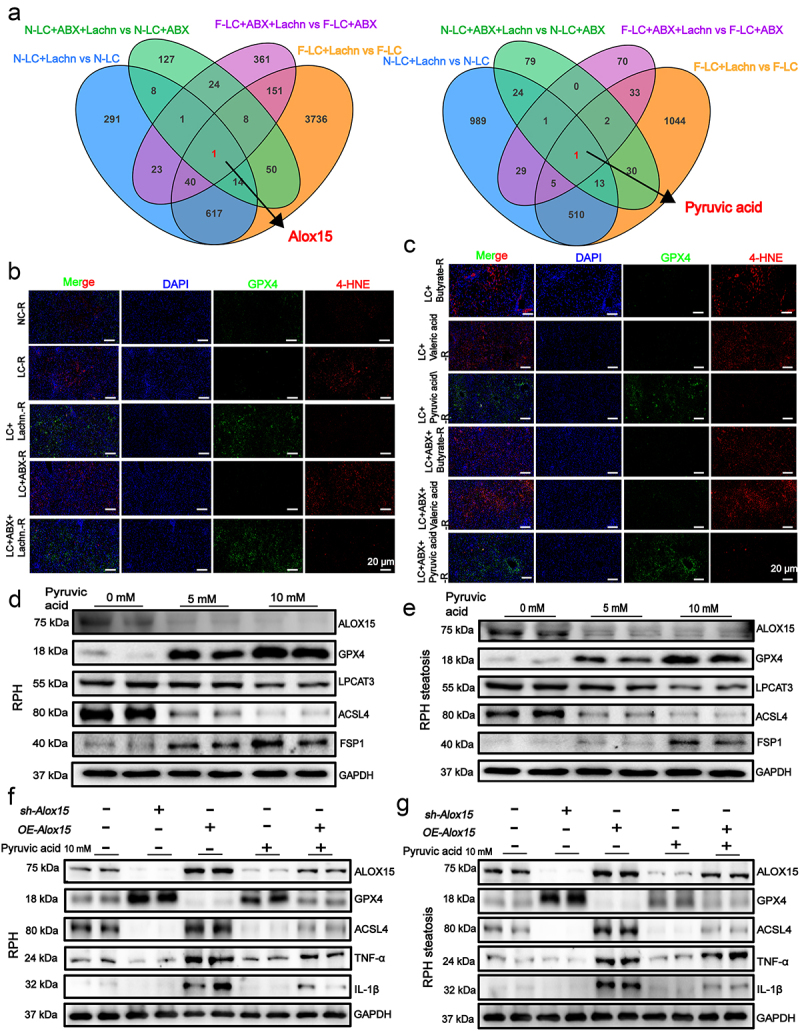
Pyruvate acid mitigates ferroptosis in transplanted liver IRI by inhibiting ALOX15 expression. (a) venn diagram showing transcriptomic and untargeted metabolomic analyses in transplanted livers from normal and steatotic donor livers to normal rats (NC-R), rats with liver cirrhosis (LC-R), lc rats treated with *Lachn*. (LC+Lachn.-R), abx-treated rats with LC (LC+ABX-R), and *Lachn*. intervention in ABX-treated rats with lc (Lc+Abx+Lachn.-R). (b,c) tissue immunofluorescence detecting ferroptosis markers in livers from steatotic donors post-IT in NC-R, LC-R, LC+Lachn.-R, LC+ABX-R, and LC+ABX+Lachn.-R (scale bar = 20 μm). (d,e) expression of ALOX15 and ferroptosis markers in IRI model rat primary hepatocytes (RPH) and steatosis rph following pyruvate intervention. (f,g) examination of Alox15 overexpression, knockdown, and pyruvate intervention in RPH and steatosis RPH, assessing inflammatory markers and ferroptosis markers (GPX4, 4-HNE) in an IRI model.

To investigate the mechanisms by which *Lachn*. alleviates IRI in LT, we performed transcriptomic and untargeted metabolomic analyses of both normal and steatotic donor livers post-transplantation. *Lachn*. intervention led to significant suppression of Alox15 expression in both normal and steatotic donor livers, while pyruvate levels were markedly elevated and inversely correlated with Alox15 expression (Figure 5a, Figure S11A, B, Figure S12A, B). *Alox15* is known to play a pivotal role in inflammation and ferroptosis by regulating lipid metabolism and inducing oxidative stress. Further validation of the relationship between Alox15 and LT-induced IRI was performed using immunofluorescence analyses of transplanted livers from NC-R, LC-R, LC+pyruvate acid-R, LC-ABX-R, and LC-ABX+pyruvate acid-R. A significant reduction in 4-hydroxy 2-nonenal (4-HNE) levels and an increase in glutathione peroxidase 4 (GPX4) expression were noted in both normal and steatotic donor livers from rats treated with *Lachn*. and pyruvate acid, with the effects being particularly pronounced in steatotic livers (Figure 5b, c, Figure S12C-E).

In vitro, pyruvate treatment in HepG2 cells, primary rat hepatocytes, and steatotic cell models confirmed its regulatory role on *Alox15* expression and ferroptosis. Western blotting revealed that pyruvate treatment (0, 5, and 10 mm) upregulated GPX4 and ferroptosis suppressor protein 1 (FSP1), while inhibiting Alox15, lysophosphatidylcholine acyltransferase 3 (LPCAT3), and acyl-CoA synthetase long chain family member 4 (ACSL4) (Figure 5d, e, Figure S13A-D). Finally, manipulation of *Alox15* expression through knockdown and overexpression in an IRI cell model confirmed the interaction between pyruvate acid and ALOX15 in regulating ferroptosis. *Alox15* knockdown significantly reduced ferroptosis and inflammatory marker expression in both normal and steatotic hepatocytes, whereas *Alox15* overexpression had the opposite effect. Notably, the biological effects of *Alox15* overexpression were mitigated by exogenous pyruvate acid treatment (Figure 5f, g, Figure S13E-G). These findings suggest that pyruvate from *Lachn*. mitigates ferroptosis and inflammation in LT-induced IRI by inhibiting Alox15 expression.

### Inhibition of ALOX15 reduces ferroptosis and mitigates IRI in steatotic donor liver

To validate the role of Alox15 in LT-induced IRI, LC rats were treated with the ALOX15 inhibitor ML351 (10 mg/kg/day) for 7 days before transplantation. H&E staining and TUNEL assay revealed a marked reduction in IRI in both normal and steatotic donor livers following ML351 treatment (Figure S14A, B). ELISA confirmed decreased serum ALT, AST, HMGB1, and IL-6 levels (Figure S14C, D). Additionally, ML351 treatment also reduced neutrophil and macrophage infiltration and suppressed ferroptosis in liver grafts (Figure S14E, F). Western blot analysis further corroborated these findings, demonstrating that ML351-mediated inhibition of ALOX15 effectively reduced ferroptosis and inflammation, thus mitigating IRI, particularly in steatotic donor livers (Figures S14G, H).

To further explore the role of ALOX15 in ferroptosis and IRI, prophylactic *Alox15* knockdown was performed in both donor livers (normal and steatotic) and LC recipients before LT. H&E staining and TUNEL assay revealed significantly reduced graft injury in both normal and steatotic donor livers after prophylactic *Alox15* knockdown. Notably, the recipient prophylactic Alox15 knockdown further attenuated graft damage (Figure S15A, B). ELISA results confirmed that *Alox15* knockdown led to a significant decrease in postoperative ALT, AST, HMGB1, and IL-6 levels (Figure S15C, D). Reduced inflammatory cell infiltration and ferroptosis were observed in liver grafts with *Alox15* knockdown, particularly in steatotic donor livers (Figures S15E, F). Western blot analysis further supported these findings, showing a substantial decrease in ferroptosis and inflammation, highlighting the enhanced protective effect in both normal and steatotic donor livers (Figure S15G, H). These results demonstrate that inhibition of *Alox15*, either through pharmacological intervention with ML351 or genetic knockdown, significantly mitigates IRI by reducing ferroptosis and inflammation. Pyruvate acid from *Lachn*. further amplifies this effect by downregulating ALOX15, providing effective protection in both normal and steatotic donor livers.

### Reduced nuclear translocation of transcription factor FOXO3 inhibits ALOX15 expression

**Figure 6.**
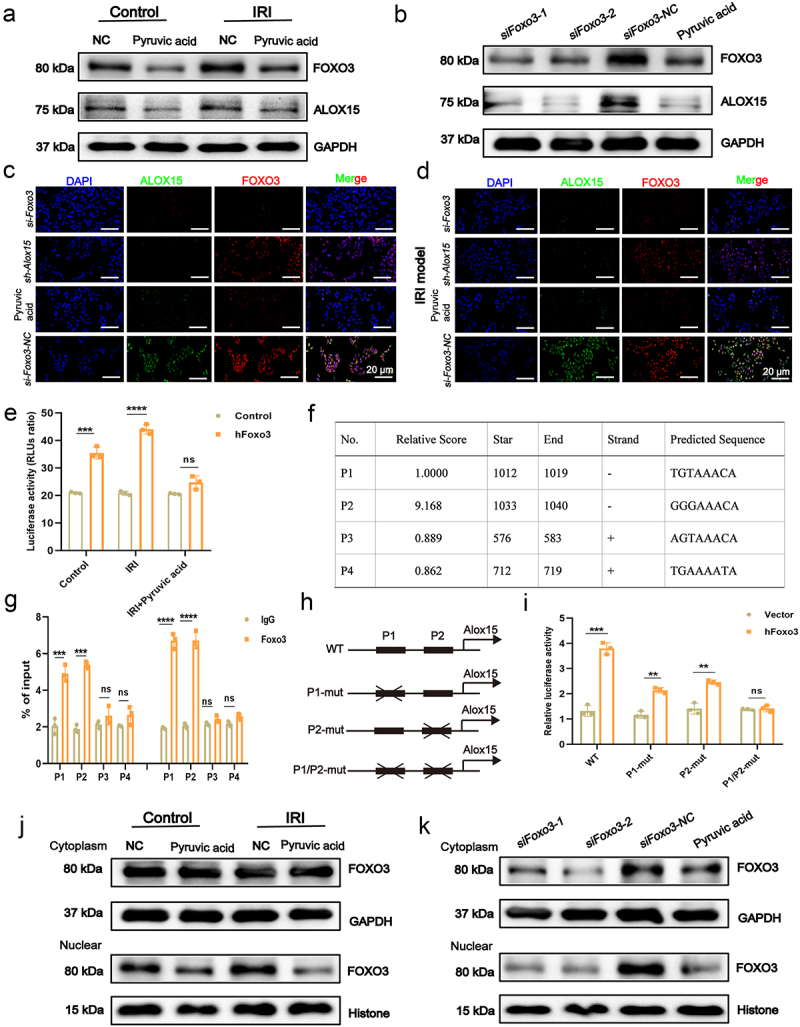
Reduced nuclear translocation of transcription factor FOXO3 inhibits ALOX15 expression. (a) Western blot analysis assessing the expression levels of FOXO3 and ALOX15 in HepG2 cells under normal culture conditions and in an IRI model following pyruvate intervention. (b) Western blot analysis to assess FOXO3 and ALOX15 expression levels following Foxo3 silencing and pyruvate intervention. (c,d) under normal culture conditions (c) and IRI model (d), examination of FOXO3 and ALOX15 expression levels in HepG2 cells with Foxo3, Alox15 knockdown, and pyruvate intervention (scale bar = 20 μm). (e) Dual-luciferase reporter assay to determine if Foxo3 can regulate Alox15 expression under normal culture conditions and in an iri model. (f) Potential binding sites of transcription factor Foxo3 on the Alox15 promoter. (g) ChIP-qPCR to verify the potential binding sites of Foxo3 on the Alox15 promoter. (H,I) Luciferase assays to revalidate the binding sites following mutation. (j) Western blot analysis of changes in cytoplasmic and nuclear FOXO3 expression levels in HepG2 cells under normal culture conditions and in an iri model with pyruvate intervention. (k) Western blot analysis to examine changes in cytoplasmic and nuclear FOXO3 expression levels following Foxo3 silencing and pyruvate intervention. **p < 0.01, ***p < 0.001, ****p < 0.0001, ns: not significant.

To elucidate the upstream regulatory mechanisms of *Alox15*, we observed coordinated changes in the transcription factors *Foxo3* and *Alox15* following intervention with *Lachn*.; and *Foxo3* exhibited an inverse relationship with pyruvate acid levels (Figure S13 G). FOXO3 reportedly plays a complex dual role in IRI, potentially promoting cell death and exacerbating IRI in the liver.^30^ To investigate the regulatory relationship between Foxo3 and Alox15, in vitro IRI cell model, pyruvate acid treatment significantly reduced the expression of both *Foxo3* and *Alox15* (Figure 6a, b). Moreover, knockdown of Foxo3 decreased ALOX15 expression in both normal and IRI conditions, whereas Alox15 knockdown did not influence FOXO3, indicating that FOXO3 regulates ALOX15 (Figure 6c, d).

To further verify whether Foxo3 regulates Alox15 expression by binding to the Alox15 promoter, a dual-luciferase reporter assay demonstrated that FOXO3 activates the Alox15 promoter, particularly under IRI conditions, an effect attenuated by pyruvate acid treatment (Figure 6e). Using the JASPAR analysis, we identified four potential FOXO3 binding sites on the *Alox15* promoter, two of which (P1 and P2) were validated using ChIP-qPCR (Figure 6f, g). Mutation of these binding sites further validated that FOXO3 directly binds and modulates Alox15 promoter activity (Figure 6h, i). To explore the mechanism by which FOXO3 regulates Alox15 expression, we analyzed the cytoplasmic and nuclear fractions of cells under both normal and IRI conditions. Pyruvic acid treatment reduced the nuclear translocation of FOXO3, and knockdown of Foxo3 also led to a significant decrease in its nuclear content (Figure 6j, k). These findings indicate that pyruvic acid derived from *Lachn*. inhibits the nuclear translocation of FOXO3, leading to the suppression of Alox15 activity. This regulatory mechanism reduces inflammation, lipid peroxidation, and ferroptosis, thereby mitigating IRI during liver transplantation.

### *Elevated* Lachn. *abundance associated with alleviated liver IRI in LT recipients*

**Figure 7.**
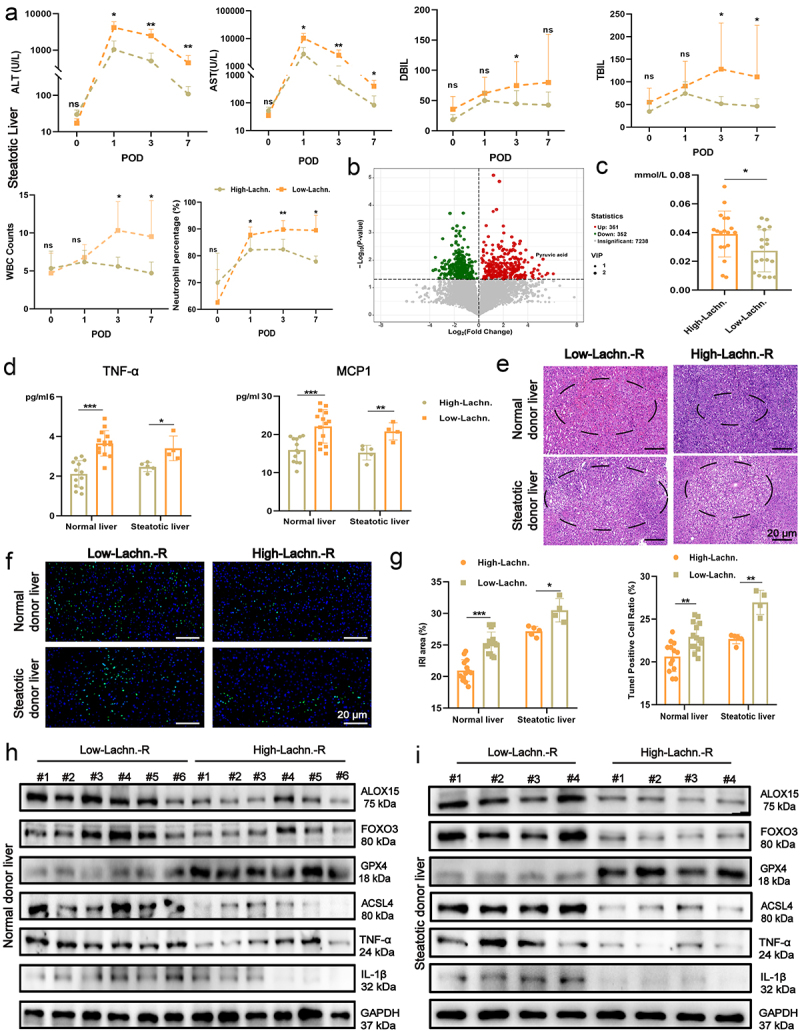
Elevated *Lachn*. abundance associated with alleviated liver IRI in LT recipients. (a) changes in alanine transaminase (alt), aspartate aminotransferase (ast), bilirubin, leukocytes, and neutrophil percentages within one week post-transplant in recipients with lc having high and low *Lachnospiraceae* abundance who received steatotic donor livers. (b) Volcano plot of differential fecal metabolites in liver cirrhosis recipients with varying *Lachn*. abundance. (c) Serum pyruvate levels in recipients with LC having different *Lachn*. abundance who received normal and steatotic donor livers. (d) serum inflammatory marker levels in recipients with LC having high and low *Lachn*. abundance who receive normal and steatotic donor livers. (e – g) Hematoxylin and eosin (H&E) and terminal deoxynucleotidyl transferase dUTP nick end labeling (tunel) assays to assess iri severity and perform semi-quantitative analysis in transplanted livers from normal and steatotic donors to recipients with different *Lachn*. abundance (scale bar = 20 μm). (h) Western blot evaluating changes in ALOX15 expression, ferroptosis markers, and inflammatory markers in transplanted livers from normal and steatotic donors to recipients with varying *Lachn*. abundance. Statistical significance was determined using one-way and two-way analysis of variance (anova). **p < 0.01, ***p < 0.001, ****p < 0.0001, ns: not significance.

To clinically validate the association between *Lachn*. abundance and IRI in transplanted livers, we collected stool, serum, clinicopathological features, and LT specimens from 36 patients with LC undergoing LT (Table S1–2). Preoperative stool samples were analyzed via metagenomic sequencing and categorized into high and low *Lachn*. abundance groups (Relative Lachn. median abundance = 0.0000435). Patients with high *Lachn*. abundance exhibited significant reductions in postoperative ALT, AST, total bilirubin (TBIL), direct bilirubin (DBIL), white blood cell count, and neutrophil percentage on days 1, 3, and 7, especially in the steatotic donor liver (Figure 7a, Figure S16A). Untargeted fecal metabolomics and serum analysis revealed elevated pyruvate acid levels in both the stool and serum of those with high *Lachn*. abundance, which positively correlated with *Lachn*. levels (Figure 7b, c, Figure S16B).

Furthermore, serum levels of inflammatory factors such as TNF-α, IL-6, CXCL2, HMGB1, and MCP-1 were significantly lower in high *Lachn*. abundance patients post-transplant, particularly in the steatotic donor liver recipients (Figure 7d, Figure S16C). H&E staining and TUNEL assay confirmed a marked reduction in IRI in both normal (high, *n* = 13; low, *n* = 14) and steatotic donor livers (high, *n* = 5; low, *n* = 4) from recipients with high *Lachn*. abundance (Figures 7e-g). These results were further supported by decreased hepatocyte ferroptosis and neutrophil and macrophage infiltration in transplanted livers from the high *Lachn*. group (Figure S16 D, E).

Protein analysis revealed significant reductions in ALOX15, FOXO3, ASCL4, TNF-α, and IL-1β levels in both normal and steatotic donor livers of recipients with high *Lachn*. abundance, suggest ing that *Lachn*. attenuates inflammation and ferroptosis by inhibiting FOXO3 and ALOX15 expression, thereby mitigating IRI, especially in steatotic donor livers (Figure 7h, i). Clinical outcomes further supported these findings, with high *Lachn*. abundance recipients experiencing fewer perioperative complications and a shorter hospital stay compared to those with low *Lachn*. abundance (Figure S16F, G). These results suggest that *Lachn*. can alleviate inflammation and IRI in LT recipients, improving clinical outcomes. Therefore, *Lachn*. has the potential to become a promising therapeutic target, particularly for the treatment of IRI in steatotic donor liver transplantation.

## Discussion

To the best of our knowledge, this study is the first to systematically examine how the gut microbiota *Lachn*. influences intestinal permeability in LC and mitigates IRI in both normal and steatotic donor livers. The primary mechanism involves the suppression of FOXO3 and ALOX15 expression in transplanted livers by pyruvate produced by *Lachn*., which subsequently reduces inflammatory responses and ferroptosis. Notably, decreased FOXO3 expression reduced its nuclear translocation and binding to the Alox15 promoter, thereby inhibiting ALOX15 activity and alleviating donor liver IRI. These findings suggest that modulating *Lachn*. abundance in the gut microbiota of patients with LC could be a promising strategy for managing IRI post-LT and expanding the donor pool.

In this study, we observed significant differences in the abundance of gut microbiota related to intestinal permeability in the fecal samples of LC rats. Specifically, there was a decrease in *Lachn*. and an increase in *Prevotella-sp*. levels. Notably, supplementation with *Lachn*. not only reduced *Prevotella-sp* abundance but also significantly improved intestinal permeability. A recent study identified an increased abundance of *Prevotella-sp*. species in individuals with rheumatoid arthritis, periodontitis, metabolic disorders, and gut dysbiosis.^31^ The pathogenicity of *Prevotella-sp*. is associated with the transition of commensal bacteria into pathogens, facilitated by factors such as adhesins, hemolysins, secretion systems, exopolysaccharides, lipopolysaccharides, proteases, quorum-sensing molecules, and antibiotic resistance, all of which can compromise the intestinal barrier.^32,33^ However, *Prevotella-sp*. is sometimes beneficial, particularly in those with fiber-rich diets, where it helps ferment plant fibers into short-chain fatty acids that promote gut health.^34^ Our findings suggest that in rats with LC, *Prevotella-sp*. abundance is associated with increased intestinal permeability, whereas *Lachn*. supplementation reduces *Prevotella-sp*. levels and improves gut barrier function. This indicates a competitive interaction between *Lachn*. and *Prevotella-sp*. in maintaining the integrity of intestinal barrier.

Notably, in this study, we identified a wide variety of metabolites produced by *Lachnospiraceae*, such as alcohols, ketones, pyrazines, short (C2–C5) and long (C > 5) chain acids, phenols, aldehydes, and 30 other compounds including the well-known metabolite butyrate.^24,35,36^ *Lachnospiraceae* and its metabolite butyrate and butyrate salts have been implicated in multiple diseases.^23,37,38^ Wang et al.^39^ found that melatonin can increase the levels of *Lachnospiraceae_NK4A136* and butyrate, alleviating hippocampal inflammation and neuronal apoptosis through the crosstalk between the toll-like receptor 4/nuclear factor kappa B and monocarboxylate transporter 1/histone deacetylase 3 signaling pathways. Similarly, Huang et al.^40^ demonstrated that increased *Lachnospiraceae* abundance and butyrate esters levels could enhance the gut barrier function and inhibit the translocation of bacteria-derived lipopolysaccharides, thereby reducing placenta-derived inflammation. However, our study is the first to show that pyruvate, not butyrate, derived from *Lachnospiraceae* exerts a protective effect in controlling hepatic IRI. Pyruvate is a 3-carbon compound found in human tissues and is physiologically used by cells as an energy substrate under anaerobic conditions; however, it has rarely been studied in the context of organ IRI. Sileri et al.^41^ reported that supplementing rats with pyruvate before liver ischemia-reperfusion (I/R) treatment reduced apoptosis and alleviated I/R injury. Additionally, Cicalese et al.^42^ evaluated the efficacy of pyruvate in organ preservation and transplantation, and found that oral pyruvate could reduce allograft rejection, inhibit perforin and granzyme-b expression, and decrease leukocyte infiltration. Nonetheless, the source of pyruvate and the mechanism by which it alleviates IRI after LT remain unclear. Our study is the first to show that pyruvate derived from *Lachn*. can inhibit Alox15 expression in the transplanted liver, suppress the inflammatory response and ferroptosis, and mitigate IRI.

Following *Lachn*. supplementation, we observed a significant reduction in *Alox15* expression in both normal and steatotic donor livers. ALOX15 is a member of the lipoxygenase family and is involved in lipid metabolism and inflammatory responses. Its expression is closely associated with tissue inflammation and oxidative stress. ALOX15 primarily induces tissue inflammation and oxidative stress via ferroptosis.^43,44^ Previous studies have highlighted the sensitivity of *ALOX15* to ferroptosis during tissue IRI. For instance, Ma et al.^45^ demonstrated that ALOX15 acts as a “burning point” during myocardial ischemia, igniting phospholipid oxidation and converting it into ferroptotic signals, thereby exacerbating myocardial cell IRI. Cai et al.^46^ confirmed that ALOX15 expression is specifically increased in injured areas and that myocardial-specific knockout of ALOX15 can reduce the expression of the ferroptosis trigger 15-HpETE, thereby alleviating I/R injury in mice. Jia et al.^47^ also confirmed that wogonin reduces lipid peroxidation and ferroptosis by regulating ALOX15 and iNOS, thereby alleviating hepatic IRI in rats and improving survival rates. In our study, *Alox15* expression significantly increased after transplantation of both normal and steatotic donor livers, which contributed to ferroptosis. However, *Lachn*. supplementation reduced ferroptosis and IRI. Additionally, we found that, during IRI, *Lachn*. inhibited the expression of the transcription factor *FOXO3*, which worked synergistically with *Alox15* to reduce injury. A previous study reported that *FOXO3* promotes IRI by inducing inflammatory responses, apoptosis, autophagy, mitosis, pyroptosis, and oxidative damage.^30^ However, *FOXO3* expression and function vary across different tissue and organ IRIs. In the kidneys, enhanced FOXO3 expression is associated with reduced apoptosis and inflammation in renal tissue, whereas in the heart, liver, and brain tissues, increased FOXO3 expression is associated with increased apoptosis and inflammatory responses.^48–51^ Our findings further support the notion that inhibiting *FOXO3* can suppress *Alox15* expression, thereby mitigating IRI in transplanted livers.

There are several limitations to our study. First, it primarily relies on a rat LT model. To enhance the reliability of our findings, validation using mouse LT models is necessary. Second, in the clinical validation of the mechanism, the sample size we included was insufficient. Considering variations in preoperative treatment regimens and gut microbiota among different populations, further studies with larger sample sizes are required. Lastly, our research is still at the experimental stage, and clinical trials are needed to facilitate its clinical translation. However, our study still has important clinical implications. In this study, we identified for the first time that pyruvate derived from *Lachn*. can suppress Foxo3 and Alox15 expression, inhibit the ferroptosis signaling pathway, and alleviate IRI in both normal and steatotic donor livers. *Lachn*. may represent a promising potential therapeutic target for preoperative strategies to mitigate IRI in LT.

In summary, this study demonstrated that supplementation with the gut bacterium *Lachn*. can restore intestinal barrier function. Additionally, *Lachn*.-derived pyruvate inhibited the expression of FOXO3 and ALOX15, resulting in reduced inflammation and ferroptosis in both normal and steatotic donor livers. Further reduction in FOXO3 expression amplified the inhibition of *Alox15* activity. Thus, preoperative maintenance of *Lachn*. abundance may represent a promising therapeutic strategy to mitigate IRI in donor livers post-LT, particularly in those with steatosis. Nonetheless, the safety and efficacy of this approach warrants further clinical investigation.

## Supplementary Material

Supplemental Material

## Data Availability

The data that support the findings of this study are available from the corresponding author upon reasonable request. The RNA and Metagenomic sequencing data generated in this study are publicly available in NCBI SRA database at PRJNA1209410, PRJNA1191191, and PRJNA1183580.

## Abbreviations

AST: Aspartate transaminase
ALOX15: Arachidonate 15-lipoxygenase
ALT: Alanine transaminase
CXCL2: C-X-C motif chemokine ligand 2
DBIL: Direct bilirubin
FD4: Fluorescein isothiocyanate dextran
FISH: Fluorescence in situ hybridization
HFD: High-fat methionine-choline-deficient diet
HFD-D: High fat diet-donor
HMGB1: High mobility group box 1
FOXO3: Forkhead box O3
GPX4: Glutathione peroxidase 4
IL6: Interleukin-6
IRI: Ischemia-reperfusion injury
Lachn: Lachnospiraceae-bacterium
LC: Liver cirrhosis
LT: Liver transplantation
LC+ABX: Antibiotic-treated liver cirrhosis
MASLD: Metabolic dysfunction-associated steatotic liver disease
MCP1: Monocyte chemoattractant protein-1
NC-R: Normal receptor
ND: Normal standard diet
ND-D: Normal diet-donor
OLT: Orthotopic liver transplantation
TBIL: Total bilirubin
TNF-α: Tumor necrosis factor-α

## References

[1] Lucey MR, Furuya KN, Foley DP, Ingelfinger JR. Liver transplantation. N Engl J Med. 2023 Nov 16. 389(20):1888–21. doi:10.1056/NEJMra2200923. 37966287

[2] Goldaracena N, Cullen JM, Kim DS, Ekser B, Halazun KJ. Expanding the donor pool for liver transplantation with marginal donors. Int J Surg. 2020 Oct. 82S:30–35. doi:10.1016/j.ijsu.2020.05.024. 32422385

[3] Durand F, Levitsky J, Cauchy F, Gilgenkrantz H, Soubrane O, Francoz C. Age and liver transplantation. J Hepatol. 2019 Apr. 70(4):745–758. doi:10.1016/j.jhep.2018.12.009. 30576701

[4] Targher G, Byrne CD, Tilg H. MASLD: a systemic metabolic disorder with cardiovascular and malignant complications. Gut. 2024 Mar 7. 73(4):691–702. doi:10.1136/gutjnl-2023-330595. 38228377

[5] Liu R, Cao H, Zhang S, Cai M, Zou T, Wang G, Zhang D, Wang X, Xu J, Deng S, et al. ZBP1-mediated apoptosis and inflammation exacerbate steatotic liver ischemia/reperfusion injury. J Clin Invest. 2024 May 14. 134(13):e180451. doi:10.1172/JCI180451. 38743492 PMC11213514

[6] Abbas SH, Ceresa CDL, Pollok JM. Steatotic donor transplant livers: preservation strategies to mitigate against ischaemia-reperfusion injury. Int J Mol Sci. 2024 Apr 24. 25(9):4648. doi:10.3390/ijms25094648. 38731866 PMC11083584

[7] Kwon Y, Gottmann P, Wang S, Tissink J, Motzler K, Sekar R, Albrecht W, Cadenas C, Hengstler JG, Schürmann A, et al. Induction of steatosis in primary human hepatocytes recapitulates key pathophysiological aspects of metabolic dysfunction-associated steatotic liver disease. J Hepatol. 2024 Jul 6. S0168-8278(24):02347–X. doi:10.1016/j.jhep.2024.06.040. 38977136

[8] Kahn J, Schemmer P. Control of ischemia-reperfusion injury in liver transplantation: potentials for increasing the donor pool. Visc Med. 2018 Dec. 34(6):444–448. doi:10.1159/000493889. 30675491 PMC6341346

[9] Cornide-Petronio ME, Negrete-Sánchez E, Mendes-Braz M, Casillas-Ramírez A, Bujaldon E, Meroño N, Martínez-Carreres L, Gracia-Sancho J, Rodés J, Jiménez-Castro MB, et al. The effect of high-mobility group box 1 in rat steatotic and nonsteatotic liver transplantation from donors after brain death. Am J Transpl. 2016 Apr. 16(4):1148–1159. doi:10.1111/ajt.13560. 26704922

[10] Dar WA, Sullivan E, Bynon JS, Eltzschig H, Ju C. Ischaemia reperfusion injury in liver transplantation: cellular and molecular mechanisms. Liver Int. 2019 May. 39(5):788–801. doi:10.1111/liv.14091. 30843314 PMC6483869

[11] Liu J, Man K. Mechanistic insight and clinical implications of ischemia/reperfusion injury post liver transplantation. Cell Mol Gastroenterol Hepatol. 2023;15(6):1463–1474. doi:10.1016/j.jcmgh.2023.03.003. 36940849 PMC10160787

[12] Yamada N, Karasawa T, Wakiya T, Sadatomo A, Ito H, Kamata R, Watanabe S, Komada T, Kimura H, Sanada Y, et al. Iron overload as a risk factor for hepatic ischemia-reperfusion injury in liver transplantation: potential role of ferroptosis. Am J Transpl. 2020 June. 20(6):1606–1618. doi:10.1111/ajt.15773. 31909544

[13] Dixon SJ, Lemberg KM, Lamprecht MR, Skouta R, Zaitsev EM, Gleason CE, Patel DN, Bauer AJ, Cantley AM, Yang WS, et al. Ferroptosis: an iron-dependent form of nonapoptotic cell death. Cell. 2012 May 25. 149(5):1060–1072. doi:10.1016/j.cell.2012.03.042. 22632970 PMC3367386

[14] Wu L, Tian X, Zuo H, Zheng W, Li X, Yuan M, Tian X, Song H. miR-124-3p delivered by exosomes from heme oxygenase-1 modified bone marrow mesenchymal stem cells inhibits ferroptosis to attenuate ischemia-reperfusion injury in steatotic grafts. J Nanobiotechnol. 2022 Apr 22. 20(1):196. doi:10.1186/s12951-022-01407-8. PMC9026664 35459211

[15] Xu J, Chen S, Liu D, Zhang Q, Luo T, Zhu J, Zhou L, Lin Y, Pan H, Chen Y, et al. Suppression of hepatocyte ferroptosis via USP19-mediated deubiquitination of SLC7A11 in ischemia-Free liver transplantation. Adv Sci (Weinh). 2024 Nov 22. e2406200. doi:10.1002/advs.202406200. 39574305 PMC11809379

[16] Ley RE, Peterson DA, Gordon JI. Ecological and evolutionary forces shaping microbial diversity in the human intestine. Cell. 2006 Feb 24. 124(4):837–848. doi:10.1016/j.cell.2006.02.017. 16497592

[17] Tang WH, Hazen SL. The contributory role of gut microbiota in cardiovascular disease. J Clin Invest. 2014 Oct. 124(10):4204–4211. doi:10.1172/JCI72331. 25271725 PMC4215189

[18] Zmora N, Suez J, Elinav E. You are what you eat: diet, health and the gut microbiota. Nat Rev Gastroenterol Hepatol. 2019 Jan. 16(1):35–56. doi:10.1038/s41575-018-0061-2. 30262901

[19] Lee J, Lee J, Kim K, Lee J, Jung Y, Hyeon JS, Seo A, Jin W, Weon B, Shin N, et al. Antibiotic-induced intestinal microbiota depletion can attenuate the acute kidney injury to chronic kidney disease transition via NADPH oxidase 2 and trimethylamine-N-oxide inhibition. Kidney Int. 2024 June. 105(6):1239–1253. doi:10.1016/j.kint.2024.01.040. 38431216

[20] Liu H, Wang J, Ding Y, Shi X, Ren H. Antibiotic pretreatment attenuates liver ischemia-reperfusion injury by farnesoid X receptor activation. Cell Death Dis. 2022 May 21. 13(5):484. doi:10.1038/s41419-022-04955-x. 35597796 PMC9124217

[21] Yang CJ, Chang HC, Sung PC, Ge MC, Tang HY, Cheng ML, Cheng HT, Chou HH, Lin CY, Lin WR, et al. Oral fecal transplantation enriches Lachnospiraceae and butyrate to mitigate acute liver injury. Cell Rep. 2024 Jan 23. 43(1):113591. doi:10.1016/j.celrep.2023.113591. 38153838

[22] Pi Y, Zuo H, Wang Y, Zheng W, Zhou H, Deng L, Song H. Oleanolic acid alleviating ischemia-reperfusion injury in rat severe steatotic liver via KEAP1/NRF2/ARE. Int Immunopharmacol. 2024 Sep 10. 138:112617. doi:10.1016/j.intimp.2024.112617. 38972213

[23] Wang L, Wang Y, Xu H, Li W. Effect of dapagliflozin on ferroptosis through the gut microbiota metabolite TMAO during myocardial ischemia-reperfusion injury in diabetes mellitus rats. Sci Rep. 2024 June 15. 14(1):13851. doi:10.1038/s41598-024-64909-5. 38879701 PMC11180094

[24] Abdugheni R, Wang WZ, Wang YJ, Du MX, Liu FL, Zhou N, Jiang CY, Wang CY, Wu L, Ma J, et al. Metabolite profiling of human-originated Lachnospiraceae at the strain level. Imeta. 2022 Oct 13. 1(4):e58. doi:10.1002/imt2.58. 38867908 PMC10989990

[25] Takeuchi T, Kameyama K, Miyauchi E, Nakanishi Y, Kanaya T, Fujii T, Kato T, Sasaki T, Tachibana N, Negishi H, et al. Fatty acid overproduction by gut commensal microbiota exacerbates obesity. Cell Metab. 2023 Feb 7. 35(2):361–375.e9. doi:10.1016/j.cmet.2022.12.013. 36652945

[26] Zhang X, Yu D, Wu D, Gao X, Shao F, Zhao M, Wang J, Ma J, Wang W, Qin X, et al. Tissue-resident Lachnospiraceae family bacteria protect against colorectal carcinogenesis by promoting tumor immune surveillance. Cell Host Microbe. 2023 Mar 8. 31(3):418–432.e8. doi:10.1016/j.chom.2023.01.013. 36893736

[27] Sun D, Bai R, Zhou W, Yao Z, Liu Y, Tang S, Ge X, Luo L, Luo C, Hu GF, et al. Angiogenin maintains gut microbe homeostasis by balancing α-proteobacteria and Lachnospiraceae. Gut. 2021 Apr. 70(4):666–676. doi:10.1136/gutjnl-2019-320135. 32843357 PMC7904960

[28] Wu Y, Ran L, Yang Y, Gao X, Peng M, Liu S, Sun L, Wan J, Wang Y, Yang K, et al. Deferasirox alleviates dss-induced ulcerative colitis in mice by inhibiting ferroptosis and improving intestinal microbiota. Life Sci. 2023 Feb 1. 314:121312. doi:10.1016/j.lfs.2022.121312. 36563842

[29] Ma Y, Li W, Niu S, Zhu X, Chu M, Wang W, Sun W, Wei X, Zhang J, Zhang Z. BzATP reverses ferroptosis-induced gut microbiota disorders in collagen-induced arthritis mice. Int Immunopharmacol. 2023 Nov. 124(Pt A):110885. doi:10.1016/j.intimp.2023.110885. 37713784

[30] Omorou M, Huang Y, Gao M, Mu C, Xu W, Han Y, Xu H. The forkhead box O3 (FOXO3): a key player in the regulation of ischemia and reperfusion injury. Cell Mol Life Sci. 2023 Mar 20. 80(4):102. doi:10.1007/s00018-023-04755-2. 36939886 PMC11072419

[31] Iljazovic A, Amend L, Galvez EJC, de Oliveira R, Strowig T. Modulation of inflammatory responses by gastrointestinal Prevotella spp. - from associations to functional studies. Int J Med Microbiol. 2021 Feb. 311(2):151472. doi:10.1016/j.ijmm.2021.151472. 33461110

[32] Sharma G, Garg N, Hasan S, Shirodkar S. Prevotella: an insight into its characteristics and associated virulence factors. Microb Pathog. 2022 Aug. 169:105673. doi:10.1016/j.micpath.2022.105673. 35843443

[33] Ley RE. Gut microbiota in 2015: prevotella in the gut: choose carefully. Nat Rev Gastroenterol Hepatol. 2016 Feb. 13(2):69–70. doi:10.1038/nrgastro.2016.4. 26828918

[34] Singh S, Giron LB, Shaikh MW, Shankaran S, Engen PA, Bogin ZR, Bambi SA, Goldman AR, Azevedo JLLC, Orgaz L, et al. Distinct intestinal microbial signatures linked to accelerated systemic and intestinal biological aging. Microbiome. 2024 Feb 22. 12(1):31. doi:10.1186/s40168-024-01758-4. 38383483 PMC10882811

[35] Tett A, Pasolli E, Masetti G, Ercolini D, Segata N. Prevotella diversity, niches and interactions with the human host. Nat Rev Microbiol. 2021 Sep. 19(9):585–599. doi:10.1038/s41579-021-00559-y. 34050328 PMC11290707

[36] Hu J, Lin S, Zheng B, Cheung PCK. Short-chain fatty acids in control of energy metabolism. Crit Rev Food Sci Nutr. 2018 May 24. 58(8):1243–1249. doi:10.1080/10408398.2016.1245650. 27786539

[37] Li Z, Zhou E, Liu C, Wicks H, Yildiz S, Razack F, Ying Z, Kooijman S, Koonen DPY, Heijink M, et al. Dietary butyrate ameliorates metabolic health associated with selective proliferation of gut Lachnospiraceae bacterium 28-4. JCI Insight. 2023 Feb 22. 8(4):e166655. doi:10.1172/jci.insight.166655. 36810253 PMC9977501

[38] He XQ, Liu D, Liu HY, Wu DT, Li HB, Zhang XS, Gan RY. Prevention of ulcerative colitis in mice by sweet tea (lithocarpus litseifolius) via the regulation of gut microbiota and butyric-acid-mediated anti-inflammatory signaling. Nutrients. 2022 May 26. 14(11):2208. doi:10.3390/nu14112208. 35684007 PMC9183097

[39] Wang X, Wang Z, Cao J, Dong Y, Chen Y. Gut microbiota-derived metabolites mediate the neuroprotective effect of melatonin in cognitive impairment induced by sleep deprivation. Microbiome. 2023 Jan 31. 11(1):17. doi:10.1186/s40168-022-01452-3. 36721179 PMC9887785

[40] Huang S, Chen J, Cui Z, Ma K, Wu D, Luo J, Li F, Xiong W, Rao S, Xiang Q, et al. Lachnospiraceae-derived butyrate mediates protection of high fermentable fiber against placental inflammation in gestational diabetes mellitus. Sci Adv. 2023 Nov 3. 9(44):eadi7337. doi:10.1126/sciadv.adi7337. 37922350 PMC10624355

[41] Cicalese L. Reviews: pyruvate in organ transplantation. JPEN J Parenter Enter Nutr. 2001 Jul. 25(4):216–218. doi:10.1177/0148607101025004216. 11434653

[42] Singh NK, Rao GN. Emerging role of 12/15-lipoxygenase (ALOX15) in human pathologies. Prog Lipid Res. 2019 Jan. 73:28–45. doi:10.1016/j.plipres.2018.11.001. 30472260 PMC6338518

[43] Li D, Lu X, Xu G, Liu S, Gong Z, Lu F, Xia X, Jiang J, Wang H, Zou F, et al. Dihydroorotate dehydrogenase regulates ferroptosis in neurons after spinal cord injury via the P53-ALOX15 signaling pathway. CNS Neurosci Ther. 2023 Jul. 29(7):1923–1939. doi:10.1111/cns.14150. 36942513 PMC10324365

[44] Wan K, Jia M, Zhang H, Lan Y, Wang S, Zhang K, Wang Z, Zhu H, Zheng X, Luo Y, et al. Electroacupuncture alleviates neuropathic pain by suppressing ferroptosis in dorsal root ganglion via SAT1/ALOX15 signaling. Mol Neurobiol. 2023 Oct. 60(10):6121–6132. doi:10.1007/s12035-023-03463-z. 37421564

[45] Ma XH, Liu JH, Liu CY, Sun WY, Duan WJ, Wang G, Kurihara H, He RR, Li YF, Chen Y, et al. ALOX15-launched PUFA-phospholipids peroxidation increases the susceptibility of ferroptosis in ischemia-induced myocardial damage. Signal Transduct Target Ther. 2022 Aug 15. 7(1):288. doi:10.1038/s41392-022-01090-z. 35970840 PMC9378747

[46] Cai W, Liu L, Shi X, Liu Y, Wang J, Fang X, Chen Z, Ai D, Zhu Y, Zhang X. Alox15/15-HpETE aggravates myocardial ischemia-reperfusion injury by promoting Cardiomyocyte Ferroptosis. Circulation. 2023 May 9. 147(19):1444–1460. doi:10.1161/CIRCULATIONAHA.122.060257. 36987924

[47] Jia D, Wu K, Luo J, Xu X, Pan W, Zhao M, Li S, Gong J, Gong J. Wogonin alleviates DCD liver ischemia/reperfusion injury by regulating ALOX15/iNOS-mediated ferroptosis. Transplantation. 2024 Jul 1. 108(12):2374–2385. doi:10.1097/TP.0000000000005123. 38946036

[48] Wang L, Niu Y, He G, Wang J. Down-regulation of lncRNA GAS5 attenuates neuronal cell injury through regulating miR-9/FOXO3 axis in cerebral ischemic stroke. RSC Adv. 2019 May 23. 9(28):16158–16166. doi:10.1039/c9ra01544b. 35521373 PMC9064354

[49] Guo X, Zhu Y, Sun Y, Li X. IL-6 accelerates renal fibrosis after acute kidney injury via DNMT1-dependent FOXO3a methylation and activation of Wnt/β-catenin pathway. Int Immunopharmacol. 2022 Aug. 109:108746. doi:10.1016/j.intimp.2022.108746. 35569307

[50] Zhang R, Li Y, Liu X, Qin S, Guo B, Chang L, Huang L, Liu S. FOXO3a-mediated long non-coding RNA LINC00261 resists cardiomyocyte hypoxia/reoxygenation injury via targeting miR23b-3p/NRF2 axis. J Cell Mol Med. 2020 Aug. 24(15):8368–8378. doi:10.1111/jcmm.15292. 32558131 PMC7412708

[51] Wang Q, Wei S, Li L, Qiu J, Zhou S, Shi C, Shi Y, Zhou H, Lu L. TGR5 deficiency aggravates hepatic ischemic/reperfusion injury via inhibiting SIRT3/FOXO3/HIF-1ɑ pathway. Cell Death Discov. 2020 Nov 1. 6(1):116. doi:10.1038/s41420-020-00347-2. 33298860 PMC7604280

