# Supplementary material for: Lachnospiraceae-bacterium alleviates ischemia-reperfusion injury in steatotic donor liver by inhibiting ferroptosis via the Foxo3-Alox15 signaling pathway
Source: Gut Microbes. 2025 Jan 30;17(1):2460543. doi: 10.1080/19490976.2025.2460543 (PMC11784649; doi:10.1080/19490976.2025.2460543)
Supplement: Supplemental Material [file KGMI_A_2460543_SM0379.zip › Supplementary Tables.docx]

***Lachnospiraceae-bacterium* alleviates ischemia-reperfusion injury in steatotic donor liver by inhibiting ferroptosis via the Foxo3-Alox15 signaling pathway**

**Supplementary Tables**

**Supplementary Table 1. Basic characteristics of High-Lachn.**

| Characteristics | High-Lachn. | | P value |
| --- | --- | --- | --- |
|  | Normal donor liver (n=13) | Steatosis donor liver (n=5) |  |
| **Gender** |  |  | 0.671 |
| Male | 9(69.2%) | 4(75.0%) |  |
| Female | 4(30.8%) | 1(25.0%) |  |
| **Age, y** | 50.0(45.0-57.0) | 50.0(36.5-57.5) | 0.581 |
| **Disease etiology** |  |  | 0.945 |
| Child A | 3(23.1%) | 1(20.0%) |  |
| Child B | 4(30.8%) | 2(40.0%) |  |
| Child C | 6(46.1%) | 2(40.0%) |  |
| **Pretransplant laboratory values** |  |  |  |
| White blood cell | 5.5(4.0-7.8) | 5.8(3.1-7.8) | 0.737 |
| Neutrophils | 3.3(1.9-5.6) | 3.3(2.0-5.1) | 0.646 |
| Neutrophil% | 70.3(60.8-76.5) | 67.6(59.7-76.9) | 0.990 |
| C-reactive protein | 25.6(7.0-46.9) | 19.1(6.8-65.9) | 0.848 |
| PCT | 3.4(1.8-8.0) | 2.8(1.9-8.3) | 0.973 |
| γ-Glutamyltransferase | 37.0(15.5-61.0) | 54.0(19.0-138.0) | 0.401 |
| AST | 45.0(32.0-56.0) | 47.0(36.0-54.1) | 0.929 |
| ALT | 29.0(17.5-39.0) | 24.0(20.5-33.4) | 0.558 |
| Total bilirubin | 29.1(14.1-46.6) | 23.1(10.8-39.3) | 0.525 |
| Direct bilirubin | 8.3(5.3-27.2) | 5.4(3.5-17.6) | 0.459 |
| Urea nitrogen | 6.3(5.5-8.6) | 7.9(3.1-10.6) | 0.979 |
| Creatinine | 66.3(60.0-75.0) | 97.6(59.6-201.7) | 0.105 |

**Supplementary Table 2. Basic characteristics of Low-Lachn.**

| Characteristics | Low-Lachn. | | P value |
| --- | --- | --- | --- |
|  | Normal donor liver (n=14) | Steatosis donor liver (n=4) |  |
| **Gender** |  |  | 0.153 |
| Male | 10(71.4%) | 2(50.0%) |  |
| Female | 4(28.6%) | 2(50.0%) |  |
| **Age, y** | 52.5(40.0-57.3) | 50.5(38.8-55.5) | 0.744 |
| **Child-Pugh grade** |  |  | 0.152 |
| Child A | 4(28.6%) | 0(0.0%) |  |
| Child B | 5(35.7%) | 2(50.0%) |  |
| Child C | 5(35.7%) | 2(50.0%) |  |
| **Pretransplant laboratory values** |  |  |  |
| White blood cell | 4.6(3.8-8.0) | 4.8(2.2-7.1） | 0.506 |
| Neutrophils | 4.6(2.8-8.5) | 2.6(1.4-4.9) | 0.217 |
| Neutrophil% | 73.0(68.3-80.4) | 60.6(51.9-75.2) | 0.077 |
| C-reactive protein | 7.7(3.5-12.0) | 11.5(5.3-17.1) | 0.931 |
| PCT | 1.8(1.0-3.3) | 3.0(1.3-3.8) | 0.864 |
| γ-Glutamyltransferase | 36.5(25.3-65.0) | 23.5(10.5-38.8) | 0.349 |
| AST | 30.9(20.5-43.8) | 35.5(27.0-46.3) | 0.940 |
| ALT | 15.0(12.8-18.3) | 17.0(12.0-22.3) | 0.776 |
| Total bilirubin | 78.9(19.9-177.9) | 169.4(31.5-633.1) | 0.087 |
| Direct bilirubin | 40.8(7.6-68.3) | 129.2(20.0-471.8) | 0.053 |
| Urea nitrogen | 9.1(6.2-10.5) | 6.9(3.4-17.8) | 0.621 |
| Creatinine | 78.8(65.9-91.7) | 68.1(45.7-122.4) | 0.927 |

**Supplementary Table 3. The shRNA sequences.**

| **si-Control** | sense 5’-UUCUUCGAACGUGUCACGUTT-3  anti-sense 5’-ACGUGACACGUUCGGAGAATT-3 |
| --- | --- |
| **si-Foxo3#1** | sense 5’-GUGUAGUACAAGGCGGACUTT-3  anti-sense 5’-AGUCCGCCUUGUACUACACTT-3 |
| **si-Foxo3 #2** | sense 5’-CCUAACAGCUCUGAACUCUTT-3  anti-sense 5’-AGAGUUCAGGCUGUUAGGTT-3 |
| **sh-Alox15 #1** | TGGGAAATCATCTATCGGTAT |
| **sh-Alox15 #2** | GCCGTCGATACATCCTATCTT |
| **AAV-shAlox15** | CTACAGGCCCAGCTGAACAAG |
| **CON207** | TTCTCCGAACGTGTCACGT |

**Supplementary Table 4. List of antibodies used in the experiment.**

| **Antibody** | **Company** | **Cat#** |
| --- | --- | --- |
| **E-cadherin** | **Abcam** | **ab231303** |
| **ZO-1** | **Abcam** | **ab221547** |
| **Occludin** | **Abcam** | **ab216327** |
| **TNF-a** | **Proteintech** | **60291-1-Ig** |
| **IL-10** | **Proteintech** | **60269-1-Ig** |
| **IL-6** | **Abcam** | **ab9324** |
| **CD11b** | **Proteintech** | **CL488-65055** |
| **CD68** | **Abcam** | **ab303565** |
| **ALOX15** | **Abcam** | **ab244205** |
| **GPX4** | **Proteintech** | **67763-1-Ig** |
| **LPCAT3** | **Proteintech** | **67882-1-Ig** |
| **ACSL4** | **Proteintech** | **22401-1-AP** |
| **FSP1** | **Proteintech** | **20886-1-AP** |
| **IL-1β** | **Proteintech·** | **26048-1-AP** |
| **FOXO3** | **Abcam** | **ab109629** |
| **Histone** | **Proteintech** | **17168-1-AP** |
| **GAPDH** | **CST** | **5174T** |

**CST: Cell Signaling Technology**

**Supplementary Table 5.** **The primer sequences for RT-qPCR.**

| **Gene** | **Forward primer (5′ - 3′)** | **Reverse primer (5′ - 3′)** |
| --- | --- | --- |
| **GAPDH (human)** | GGCAGAGATGATGACCCTTTT | AGATCCCTCCAAAATCAAGTGG |
| **Gapdh (rat)** | GTCCATGCCATCACTGCCACTC | CGCCTGCTTCACCACCTTCTTG |
| **E-cadherin (human)** | ACAACGACCCAACCCAAGAATCTATC | GCAAGAGCAGCAGAATCAGAATTAGC |
| **Occludin (human)** | GCCTCTTGAAAGTCCACCTCCTTAC | TCTGTATAGCCTCCGTAGCCATAGC |
| **Zo-1 (human)** | GGCGGATGGTGCTACAAGTGATG | AGGCTCAGAGGACCGTGTAATGG |
| **E-cadherin (rat)** | GGACAGAGAAGCCATTGACAAGTACC | GCAGCCTGAACCACCAGAGTATATG |
| **Occludin (rat)** | CAGCAACGATAACCTAGAGACACCTTC | TATTCATCAGCAGCAGCCATGTACTC |
| **Zo-1 (rat)** | AACATATCACCACAGACATCCAACCAG | GAACAGAAGACCACCAACCACTCTC |
| **Tnf-a (rat)** | CTGGCGTGTTCATCCGTTCTCTAC | ACTACTTCAGCGTCTCGTGTGTTTC |
| **Il-10 (rat)** | AGGACTTTAAGGGTTACTTGGGTTGC | CTTCACCTGCTCCACTGCCTTG |
| **Il-6 (rat)** | CTGGTCTTCTGGAGTTCCGTTTCTAC | GATGAGTTGGATGGTCTTGGTCCTTAG |
| **Cxcl2 (rat)** | AAGGGTTGACTTCAAGAACATCCAGAG | ACATCAGGTACGATCCAGGCTTCC |
| **Hmgb1 (rat)** | ACAACACTGCTGCGGATGACAAG | GTCTTCCTCCTCTTCCTCCTCTTCC |
| **Mcp1 (rat)** | TTGTGTTGGTGTTGGACGAGATGG | GGTGTAAGGTGGGAAGTTCAGTAGC |

**Supplementary Table 6.** **The primer sequences for ChIP-qPCR.**

| **Gene** | **Forward primer (5′ - 3′)** | **Reverse primer (5′ - 3′)** |
| --- | --- | --- |
| **Alox15 CHIP PRIMER 1** | GCAGTGAGCCGAGATCACAC | CAGGGACTACACACCCAGCA |
| **Alox15 CHIP PRIMER 2** | TGACACCCACAAACCCTGAC | CCCACCTCAGTCTCCCAAAG |
| **Alox15 CHIP PRIMER 3** | TGACACCCACAAACCCTGAC | CTTGTGATCCACCCACCTCA |
| **Alox15 CHIP PRIMER 4** | GGCCAAGAGGAGGGAGAAAC | CTCACTGCAACCTCCACCTG |
