# Supplementary material for: Lachnospiraceae-bacterium alleviates ischemia-reperfusion injury in steatotic donor liver by inhibiting ferroptosis via the Foxo3-Alox15 signaling pathway
Source: Gut Microbes. 2025 Jan 30;17(1):2460543. doi: 10.1080/19490976.2025.2460543 (PMC11784649; doi:10.1080/19490976.2025.2460543)
Supplement: Supplemental Material [file KGMI_A_2460543_SM0379.zip › Supplementary Methods.docx]

***Lachnospiraceae-bacterium* alleviates ischemia-reperfusion injury in steatotic donor liver by inhibiting ferroptosis via the Foxo3-Alox15 signaling pathway**

**Materials and Methods**

**1. Collection of clinicopathological characteristics, stool, serum, and liver tissue**

Upon admission, comprehensive clinical data, including patient age, sex, and detailed disease history, were systematically recorded for all study participants. Stool specimens were collected using sterile techniques into containers and immediately stored in liquid nitrogen. Blood biochemical examination results were collected the day before surgery and one week after surgery, and followed up for 1 year. During liver transplant procedures, samples of portal vein blood and liver tissue were obtained two hours following the reperfusion of the donated liver. Serum samples were centrifuged at 4000 rpm for 10 minutes and along with tissue specimens, were either cryopreserved in liquid nitrogen or chemically fixed in formalin for histological analysis. All donated livers were obtained through the China Organ Transplant Response System between August 2022 and August 2024. Steatotic donor liver was independently diagnosed by two pathologists using a criterion of more than 5% macrovesicular steatosis. A total of 36 patients were enrolled in this study, all of whom provided informed consent. The study involving human subjects was reviewed and approved by the Ethics Committee and Institutional Review Board of Wuhan Union Hospital (No. UHCT-IEC-SOP-007-02-05).

**2. Cell culture**

HepG2 cells and primary rat hepatocytes were cultured in Dulbecco’s Modified Eagle Medium (DMEM; PM150210, Procell) supplemented with 10% (v/v) fetal bovine serum (FBS, No. 10099141, Gibco) and 100 U/mL penicillin/streptomycin (No. 15-140-122, Gibco). All cultures were maintained at 37°C in a 5% CO_2_ atmosphere.

**2.1 Primary hepatocyte isolation**

Primary rat hepatocytes (PRH) were isolated under pentobarbital sodium anesthesia (50 mg/kg). Livers were perfused via the portal vein with Liver Perfusion Medium (17701-038, Life Technologies) followed by Liver Digest Medium (17703-034, Life Technologies), each at a flow rate of 2 mL/min for 5 min. Following digestion, liver tissues were excised, minced, and filtered through a 70 μm cell strainer (352350, Falcon). Hepatocytes were then collected by centrifugation at 50 × g for 3 min, repeated three times.

**2.2 Cell hypoxia reoxygenation (H/R) model.**

Cells were initially cultured in DMEM supplemented with 10% FBS and 4500 mg/L glucose to promote adherence. For hypoxia induction, cells were washed with PBS, and the medium was replaced with glucose-free DMEM devoid of FBS, then transferred to a hypoxic incubator (5% CO_2_, 94% N_2_, 1% O_2_). After 10 hours, cells were washed again and the medium was switched to glucose-replete DMEM with 10% FBS for reoxygenation 6 hours. Cells were subsequently analyzed at designated time points.

**2.3 Cell transfection and reagents**

Overexpression plasmids for Alox15 and lentiviral shRNA, along with AAV8-shRNA particles, were acquired from GeneChem Co. (Shanghai, China). siRNA was sourced from GenePharma (Shanghai, China). Transfections were conducted using Lipofectamine 2000 (No.11668019, Invitrogen) in Opti-MEM (No.31985070, Gibco). Six hours post-transfection, Opti-MEM was replaced with fresh DMEM containing 10% FBS. Lentiviral transfections were carried out as per the manufacturer’s protocol, seeding 5000 cells per well in 96-well plates. Lentiviral particles were added the following day with 10 mg/mL polybrene (C0351, Beyotime), and cells were subsequently selected with 5 μg/mL puromycin (CL13900, MedChem Express). For in vivo knockdown of Alox15, an AAV8 vector (pAAV-U6-shRNA-WPRE) was administered to rats via tail vein injection (6 × 10^11 viral genomes in 600 μL). The sequences of shRNAs are provided in Supplementary Table 3.

**2.4 Construction of hepatocyte steatosis cell model**

HepG2 cells and primary hepatocytes were cultured for 48 hours before being treated with a free fatty acid (FFA) mixture (oleic acid to palmitic acid ratio of 2:1) at 1.5 mmol/L to induce steatosis over 24 hours. Lipid accumulation within hepatocytes was assessed using Oil Red O staining.

**3. Immunoblotting and immunofluorescence**

Tissues or cells were lysed on ice using a radioimmunoprecipitation assay buffer containing 1% protease and phosphatase inhibitors. The lysates were centrifuged at 4°C, 12,000 rpm for 15 minutes, and the supernatants were collected. Protein concentrations were determined using a bicinchoninic acid assay. Proteins were mixed with 5× loading buffer and heated at 95°C for 10 minutes before being subjected to Western blot analysis. For immunohistochemistry (IHC) assays, paraffin-embedded specimens were sectioned and treated with hydrogen peroxide to block endogenous peroxidase activity and with a blocking solution to reduce non-specific antigen binding. Primary antibodies were applied and incubated overnight at 4°C, followed by incubation with secondary antibodies. Detection was achieved using a DAB staining kit (DA1016, Solarbio). Immunofluorescence of tissues or cells was conducted similarly, except the samples were incubated in dark conditions and examined using either a fluorescence or confocal microscope following 4,6-diamidino-2-phenylindole (DAPI) staining. Detailed information regarding the antibodies used in this experiment is provided in the Supplementary Table 4.

**4. RNA extraction and quantitative real-time polymerase chain reaction (qRT-PCR).**

Total RNA was extracted from both tissues and cells utilizing TRIzol reagent (No.15596026, Invitrogen) along with the RNApure Tissue/Cell Kit (CW0584S, CWBIO). The RNA concentrations were quantified using a spectrophotometer. Subsequently, 1 μg of each RNA sample was reverse transcribed into cDNA employing the Reverse Transcription Kit (R323-01, Vazyme). Quantitative real-time PCR (qRT-PCR) analyses were conducted using the Super SYBR Green Kit (R223-01, Vazyme). Each sample was assessed in triplicate, and the mean values were used for subsequent data analysis. The primer sequences employed for amplifying these genes are provided in Supplementary Table 5.

**5. Enzyme-linked immunosorbent assay (ELISA).**

Serum concentrations of ALT, AST, CXCL2, HMGB1, and MCP-1 were quantified using enzyme-linked immunosorbent assays (ELISA; ELISA LAB, Wuhan) following the manufacturer’s protocols. Each sample was assayed in quintuplicate. Absorbance (OD) at 450 nm was measured using an automated enzyme labeler. Concentrations of ALT, AST, CXCL2, HMGB1, and MCP-1 were calculated from standard curves. The detailed experimental procedure was carried out according to the protocol described in the experimental manual.

**6. Tissue section staining and analysis**

**6.1 Hematoxylin and eosin (H&E) stain**

Hematoxylin and Eosin (H&E) staining was conducted using a standard protocol. Tissue sections were dewaxed with xylene and dehydrated through a graded series of ethanol. Staining involved immersing the tissues in hematoxylin for 20 minutes at room temperature followed by eosin for 2 minutes. Sections were then dehydrated again in ethanol and sealed with a neutral gel mount. Each section was meticulously washed with distilled water between steps. For each tissue section, five random fields were selected to quantify the area of damage. The sample size for each experimental group was n=5.

**6.2 Oil red O stain**

Oil Red O staining was adapted to evaluate neutral lipids and lipid droplet morphology in tissues. Tissues from mice or rats of varying genotypes were fixed in 4% paraformaldehyde, rinsed in PBS, and incubated overnight at 4 °C in 30% sucrose. Following this, tissues were embedded in a 2:1 mixture of 30% sucrose and Tissue-Tek OCT compound and frozen. Sections prepared from these frozen tissues were stained in a freshly prepared 1% Oil Red O solution for 10 minutes and counterstained with hematoxylin. They were then rinsed under running tap water for 30 minutes. Photomicrographs of the stained sections were captured using a light microscope.

**6.3 TUNEL stain**

Liver tissues were fixed in 4% paraformaldehyde and processed for paraffin embedding. The detection of apoptotic cells featuring DNA fragmentation was performed using the TUNEL assay with the In Situ Cell Death Detection Kit, POD (Roche), according to the manufacturer's instructions. Quantitative analysis of TUNEL positivity was conducted using Fiji software. The sample size for each experimental group was n=5.

**7. Transmission electron microscopy examination**

Colonic and rectal tissues from rats were collected and cut into tissue blocks not exceeding 1 × 1 × 1 mm³. These samples were immediately fixed in electron microscopy fixative for 2-4 hours. The tissues were then rinsed three times with PBS, each rinse lasting 15 minutes. Afterward, they were post-fixed in 1% osmium tetroxide in 0.1M phosphate-buffered saline (PBS) and rinsed again. The tissues were subsequently dehydrated, infiltrated, and embedded for sectioning. The sections were then stained with uranyl acetate and lead citrate, followed by air drying at room temperature overnight. Finally, the samples were examined using transmission electron microscopy, and images were captured. The sample size for each experimental group was n=5.

**8. Intestinal Permeability Assay**

Liver cirrhosis rats, as well as those subjected to antibiotic or *Lachnospiraceae* intervention, were administered a single dose of FITC-dextran (FITC-D, No.46944, Sigma Aldrich, 600 mg/kg). The control group received PBS. Four hours post-administration, rats were anesthetized with isoflurane (5% for induction, 2% for maintenance). 1 mL of blood was collected from the inferior vena cava, and plasma was separated and diluted 1:1. The samples were analyzed using a fluorescence spectrophotometer, with excitation/emission wavelengths of 485 nm/530 nm (for FITC detection). The signal intensity for each rat was measured in triplicate, and the mean value was calculated. Each group contained five rats, and plasma dextran concentrations were calculated using a standard curve for each rat.

**9. Fluorescence in situ hybridization (FISH) assay**

The obtained colorectal tissues were embedded, sectioned, and deparaffinized. The sections were then digested with proteinase K, followed by probe hybridization. Two probes were diluted in a hybridization buffer at a ratio of 1:1:100 to prepare the hybridization solution, which was then applied to the tissue sections. The sections were incubated in a humidified chamber at 42°C overnight for hybridization. After hybridization, the sections were washed and stained with DAPI to visualize the nuclei, followed by incubation in the dark for 3–10 minutes. Finally, the sections were mounted and examined using a fluorescence microscope to capture images. The probe sequences used were *Lachnospiraceae* bacterium: UCUUCCCUGCUGAUAGA and Prevotella spp.: GGUCUGCAACCCGACC.

**10. *Lachnospiraceae****-****bacterium* fecal microbiota transplantation, cell-free supernatant extraction, and co-culture model**

*Lachnospiraceae-bacterium* (BNCC-354474) were activated and cultivated on agar plates within an anaerobic chamber equipped with anaerobic gas packs. Bacterial colonies were collected using sterile inoculation loops to prepare a bacterial suspension. The suspension was then coated with 20% glycerol and administered to rats via oral gavage at a concentration of 1×10^9 CFU/ml, twice a week for a total of 3 weeks. To prepare the cell-free supernatant, *Lach.* were inoculated into Gifu Anaerobic Medium (GAM, No.HB8518-1, hopebio) broth and cultured under anaerobic conditions for 24 hours. The culture was then centrifuged at 5000 rpm for 10 minutes. The supernatant was collected and filtered through a 0.22 µm sterile filter. The filtered conditioned medium was subsequently used for co-culture with cells.

**11. Antibiotic cocktail (ABX) model and microbial metabolite intervention**

For the rat microbiota transplantation model, cirrhotic rats were administered an antibiotic cocktail consisting of ampicillin (1 g/L, No.HY-B0522, MedChemExpress), vancomycin (0.5 g/L, No.HY-B0671, MedChemExpress), neomycin (1 g/L, No.S2568, Selleck), and metronidazole (1 g/L, No.HY-B0318, Selleck) in sterile water for 30 days. For the metabolite intervention in rats, the animals were orally gavaged with 500 mg/kg of butyrate (No.B103500, Sigma), valerate (No.240370, Sigma), or pyruvate (No.107360, Sigma) daily to minimize variations due to individual differences in daily water intake. The control group was gavaged with 500 mg/kg of sodium chloride for a duration of 2 weeks. The sample size of rats in each group was at least five.

**12. Dual luciferase reporting assay and chromatin immunoprecipitation (ChIP)-qPCR**

We constructed and confirmed the sequences of the pGL4.31-hAlox15 promoter-Luc2P-SV40-hRluc and pcDNA3.1-hFoxo3 vectors through DNA sequencing. Cells were transfected with specified plasmids using Lipofectamine 2000, following the manufacturer’s guidelines. Forty-eight hours after transfection, cells were harvested and analyzed with the Dual-Luciferase Reporter Gene Assay kit (JKR23008, GENE CREATE). Chromatin immunoprecipitation assays were conducted according to the protocols provided with the ChIP-IT Express Kit, ChIP-IT Express Shearing Kit, and ChIP-IT Protein G Magnetic Beads (Active Motif, cat# 53008, 53032, 53014). The primers used for ChIP-qPCR are listed in Supplementary Table 6.

**13.** **Transcriptome profiling**

Total RNA was extracted from gastrointestinal tissue samples employing the Trizol method, followed by a rigorous assessment of RNA integrity. Recognizing that the majority of eukaryotic mRNAs feature a polyA tail, we enriched for polyA-tailed mRNA using Oligo(dT) magnetic beads. This mRNA was then purified and reverse transcribed to synthesize first-strand cDNA, with subsequent synthesis of second-strand cDNA. We then executed end-repair processes and dA-tailing, followed by adapter ligation to produce a library with insert fragments ranging from 250 to 350 bp. These ligation products underwent PCR amplification, and the resultant products were purified to finalize the library construction. After rigorous quality control, different libraries were pooled based on their effective concentrations and the desired data output, and sequenced on an Illumina platform to yield 150 bp paired-end reads. The sequencing relied on the Sequencing by Synthesis (SBS) principle. During this process, a mix of fluorescently labeled dNTPs, DNA polymerase, and adapter primers was introduced into the flow cell. As the complementary strand was extended in each sequencing cluster, the incorporation of each labeled dNTP triggered the emission of a specific fluorescence signal. These signals were captured by the sequencer, with data subsequently converted into sequencing peaks via proprietary software, enabling precise determination of sequence information for target fragments. Data analysis was conducted following sequencing to interpret the results.

**14. Metagenomic sequencing**

Total genomic DNA was extracted from 0.5 g of fecal material utilizing the PF MagO-Bind Stool DNA Kit (Omega Bio-tek, Norcross, GA, USA) per the manufacturer's protocol. DNA concentration and purity were quantified via a TBS-380 fluorometer and a NanoDrop 2000 spectrophotometer, respectively. Quality assessments of the DNA were conducted using 1% agarose gel electrophoresis. Subsequent fragmentation of the DNA was achieved using a Covaris M220 instrument (Gene Company Limited, China), generating fragments averaging 400 base pairs (bp). Paired-end libraries were prepared with the NEXTFLEX Rapid DNA-Seq Kit (Bioo Scientific, Austin, TX, USA). Sequencing was executed on an Illumina NovaSeq 6000 platform (Illumina Inc., San Diego, CA, USA) at Majorbio Bio-Pharm Technology Co., Ltd (Shanghai, China), utilizing the NovaSeq 6000 S4 Reagent Kit in accordance with the manufacturer’s guidelines. Data analysis was performed on the Majorbio Cloud Platform ([www.majorbio.com](http://www.majorbio.com)). Initially, raw sequencing reads were refined by removing adapters and discarding low-quality reads via the fastp tool. The quality-filtered reads were then assembled; contigs ≥300 bp were selected for the final assembly. A non-redundant gene catalog was developed using CD-HIT, and gene abundance in each sample was quantified using SOAPaligner. These non-redundant genes were aligned against the NCBI NR database employing DIAMOND to facilitate optimal classification and functional annotation. Differential analysis across taxonomic, functional, and gene levels was carried out using the Kruskal-Wallis test.

**15. untargeted metabolomics**

Samples stored at -80°C were thawed on ice. The samples were ground in liquid nitrogen. A volume of 400 μL of solvent (methanol = 7:3, v/v) was added to 20 mg of the sample, and the mixture was vortexed at 1500 rpm for 5 minutes. After standing on ice for 15 minutes, the sample was centrifuged at 12,000 rpm for 10 minutes at 4°C. The supernatant was collected and kept at -20°C for 30 minutes, followed by centrifugation at 12,000 rpm for 3 minutes. A 200 μL aliquot of the upper layer was then collected for analysis.

One aliquot was analyzed under positive ion conditions and was eluted from a T3 column (Waters ACQUITY Premier HSS T3 Column, 1.8 μm, 2.1 mm × 100 mm) using 0.1% formic acid in water as solvent A and 0.1% formic acid in acetonitrile as solvent B. The following gradient was applied: 5% to 20% solvent B over 2 minutes, increased to 60% over the next 3 minutes, further increased to 99% over 1 minute, held for 1.5 minutes, then returned to 5% solvent B within 0.1 minutes, and held for 2.4 minutes. The analytical conditions were as follows: column temperature, 40°C; flow rate, 0.4 mL/min; injection volume, 4 μL. Another aliquot was analyzed under negative ion conditions using the same elution gradient as in the positive mode.

**16. Statistics**

All data are presented as mean ± SD. Statistical analyses were conducted using GraphPad Prism version 8.4.1. Comparisons between two groups were made using unpaired, two-tailed Student's t-tests, one-way ANOVA were applied for multiple group comparisons against specified controls. For datasets involving more than one variable, two-way ANOVA was employed for multiple comparisons. In cases of non-normal distributions, the Mann-Whitney U test was used for two-group comparisons, and the Kruskal-Wallis test was utilized for multiple comparisons. Survival analysis was performed using Kaplan-Meier method. Significance levels were set at *P < 0.05, **P < 0.01, ***P < 0.001; non-significant results were denoted as NS. P-values less than 0.05 were considered statistically significant.
