# Supplementary material for: Lachnospiraceae-bacterium alleviates ischemia-reperfusion injury in steatotic donor liver by inhibiting ferroptosis via the Foxo3-Alox15 signaling pathway
Source: Gut Microbes. 2025 Jan 30;17(1):2460543. doi: 10.1080/19490976.2025.2460543 (PMC11784649; doi:10.1080/19490976.2025.2460543)
Supplement: Supplemental Material [file KGMI_A_2460543_SM0379.zip › Supplementary Figure legends.docx]

***Lachnospiraceae-bacterium* alleviates ischemia-reperfusion injury in steatotic donor liver by inhibiting ferroptosis via the Foxo3-Alox15 signaling pathway**

**Supplementary Figure Legends**

**Figure S1. Increased intestinal permeability in rats with liver cirrhosis. (A)** H&E and Masson’s trichrome staining of liver sections from normal and liver cirrhosis (LC) rats (Scale bar = 50 μm). **(B)** Detection of tight junctions in colon cells of normal and LC rats. **(C)** Electron microscopy of tight junctions in colon epithelium from normal and LC rats (Scale bar = 2 μm). **(D)** Fluorescein isothiocyanate (FITC)-dextran (FD4) intestinal permeability assay in normal and LC rats. **(E–G)** Fecal metagenomic sequencing analyses including alpha-diversity (E), beta-diversity (F), and principal coordinate analysis (G) in normal and LC rats. **(H,I)** Changes in fecal microbiota abundance at the species level in normal and LC rats. Statistical significance was determined using unpaired Student’s t-test and Wilcoxon rank-sum test. ***P*<0.01.

**Figure S2. Decreased abundance of *Lachn.* in rats with liver cirrhosis. (A)** Fluorescence in situ hybridization (FISH) analysis of *Lachn.* and *Prevotella* in the intestines of normal and liver cirrhosis (LC) rats (Scale bar = 20 μm). **(B)** Correlation analysis of *Lachn.* and *Prevotella-sp.* at genus and species levels with alanine transaminase (ALT) and aspartate aminotransferase (AST) in normal and LC rats. **(C)** Functional analysis of intestinal microbiota in normal and LC rats. **(D)** Volcano plot of differential non-targeted metabolomics in feces of normal and LC rats. **(E)** Functional analysis of differential metabolites in feces of normal and LC rats. **(F)** Correlation analysis of *Lachn.* with protective intestinal differential metabolites (pyruvic, succinic, and valeric acid).

**Figure S3. *Lachn.* improves intestinal permeability in rats with liver cirrhosis. (A)** Oil Red O staining of normal diet donor (ND-D) and high-fat diet donor (HFD-D) livers (Scale bar = 50 μm). **(B–F)** Analysis of gut microbiota alpha (B) and beta diversity (C), principal component analysis (D), changes in microbial abundance (E, F), and functional analysis in normal (NC-R), liver cirrhosis rats (LC-R), and LC rats treated with *Lachn.* (LC+Lachn.-R). **(F)** Assessment of changes in tight junctions in the intestines of NC-R, LC-R, and LC+Lachn.-R using qPCR. **(G)** Changes in alanine transaminase (ALT) and aspartate aminotransferase (AST) levels post-transplantation of ND-D and HFD-D livers in NC-R, LC-R, and LC+Lachn.-R. Statistical significance was determined using unpaired Student’s t-test. ***P*<0.01, ****P* <0.001, *****P*<0.0001, ns: not significant.

**Figure S4. *Lachn.* alleviates steatotic donor liver IRI in rats with liver cirrhosis. (A,B)** qPCR (A) and western blot (B) analysis of liver factors in normal (NC-R), liver cirrhosis rats (LC-R), and LC rats treated with *Lachn.* (LC+Lachn.-R) post-transplantation with normal diet donor (ND-D) and high-fat diet donor (HFD-D) livers. **(C)** qPCR and enzyme-linked immunosorbent assay (ELISA) analysis of immune-related inflammatory factors in the transplanted liver and portal vein serum post-transplantation with ND-D and HFD-D livers in NC-R, LC-R, and LC+Lachn.-R. **(D)** Immunofluorescence analysis of neutrophil and macrophage infiltration in the transplanted liver post-transplantation with ND-D and HFD-D livers in NC-R, LC-R, and LC+Lachn.-R (Scale bar = 50 μm). Statistical significance was determined using unpaired Student’s t-test. **P*<0.05, ***P*<0.01, ****P*<0.001.

**Figure S5. Validation of the effect of *Lachn.* improves intestinal permeability in antibiotic-treated liver cirrhosis (LC+ABX) rats. (A–E)** Analysis of alpha diversity (A), beta diversity (B), principal component analysis (C), changes in microbial abundance (D), and functional analysis (E) of the intestinal microbiome in liver cirrhosis+ABX rats (LC+ABX-R), and *Lachn.* intervention in ABX-treated rats with LC (LC+ABX+Lachn.-R). **(F)** assessment of changes in tight junctions in the intestines of LC-R, LC+ABX-R, and LC+ABX+Lachn.-R using qPCR. **(G)** Changes in alanine transaminase (ALT) and aspartate aminotransferase (AST) levels post-transplantation of normal diet donor (ND-D) and high-fat diet donor (HFD-D) livers in LC-R, LC+ABX-R, and LC+ABX+Lachn.-R. **(H)** Semi-quantitative terminal deoxynucleotidyl transferase dUTP nick end labeling (TUNEL) analysis of liver IRI following transplantation of ND-D and HFD-D livers in LC-R, LC+ABX-R, and LC+ABX+Lachn.-R. Statistical significance was determined using unpaired Student’s t-test. **P*<0.05, ***P*<0.01, ****P*<0.001, ns: not significant.

**Figure S6. Validation of the mitigating effect of *Lachn.* in steatotic donor LT IRI in antibiotic-treated liver cirrhosis (LC+ABX) rats. (A,B)** qPCR (A) and Western blot (B) analyses of inflammatory markers in the liver following transplantation of ND-D and HFD-D livers in LC-R, LC+ABX-R, and LC+ABX+Lachn-R rats. **(C)** qPCR and ELISA assessment of immune-related inflammatory markers in the transplant liver and portal vein serum following transplantation of ND-D and HFD-D livers in in LC-R, LC+ABX-R, and LC+ABX+Lachn-R rats. **(D)** Immunofluorescence analysis of neutrophil and macrophage infiltration in the transplant liver following transplantation of ND-D and HFD-D livers in in LC-R, LC+ABX-R, and LC+ABX+Lachn-R rats (Scale bar = 50 μm). Statistical significance was determined by unpaired Student t test. **P* <0.05, ***P* <0.01, ****P* <0.001, ns: not significant.

**Figure S7. Pyruvate acid from *Lachn.* enhances intestinal permeability. (A)** Heatmap of differential metabolic products in feces from rats with liver cirrhosis+Lachn. (LC+Lachn.), liver cirrhosis (LC), ABX-treated liver cirrhosis (LC+ABX), and ABX-treated liver cirrhosis+Lachn (LC+ABX+Lachn.). **(B)** qPCR analysis of changes in gene expression of tight junctions in the intestines of liver cirrhosis rats after intervention with Pyruvic, Valeric, and Butyrate acids. **(C)** ALT and AST levels in liver cirrhosis rats following transplantation of ND-D and HFD-D liver after intervention with Pyruvic, Valeric, and Butyrate acids. **(D)** Semi-quantitative TUNEL analysis of liver IRI in liver cirrhosis rats post-intervention with Pyruvic, Valeric, and Butyrate acids, following transplantation. **(E)** qPCR analysis of inflammatory markers in the liver post-transplantation of ND-D and HFD-D liver in LC rats treated with Pyruvic, Valeric, and Butyrate acids. Statistical significance was determined by unpaired Student t test. **P* <0.05, ***P* <0.01, ****P* <0.001, *****P* <0.0001, ns: not significant.

**Figure S8. Pyruvate acid from *Lachn.* mitigates steatotic donor liver LT IRI. (A)** Western blot analysis of inflammatory markers in the liver of liver cirrhosis rats post-intervention with Pyruvic, Valeric, and Butyrate acids, following transplantation of ND-D and HFD-D livers. **(B,C)** qPCR (B) and ELISA (C) assessment of immune-related inflammatory markers in the liver and portal vein serum of liver cirrhosis rats treated with Pyruvic, Valeric, and Butyrate acids, post-transplantation. **(D)** Immunofluorescence analysis of neutrophil and macrophage infiltration in the transplant liver of liver cirrhosis rats treated with Pyruvic, Valeric, and Butyrate acids, following transplantation of ND-D and HFD-D livers (Scale bar = 50 μm). Statistical significance was determined by unpaired Student t test. **P* <0.05, ***P* <0.01, ****P* <0.001, *****P* <0.0001, ns: not significant.

**Figure S9. Validation of improved intestinal permeability by pyruvate acid from *Lachn.* in rats with liver cirrhosis. (A)** FISH analysis to examine changes in *Lachn.* and *Prevotella-sp.* in the colon of LC+ABX rats after intervention with Pyruvic, Valeric, and Butyrate acids (Scale bar = 50 μm). **(B)** Western blot analysis of tight junction protein expression in the colonic epithelial cells post-intervention with Pyruvic, Valeric, and Butyrate acids. **(C)** qPCR assessment of RNA levels of tight junction marker proteins in LC+ABX rats after intervention with Pyruvic, Valeric, and Butyrate acids. **(D)** ALT and AST levels measurement in LC+ABX rats after intervention with Pyruvic, Valeric, and Butyrate acids, following transplantation of ND-D and HFD-D livers. **(E)** qPCR analysis of inflammatory markers in the liver of LC+ABX rats post-intervention with Pyruvic, Valeric, and Butyrate acids, following transplantation. Statistical significance was determined by unpaired Student t test. *P <0.05, **P <0.01, ***P <0.001, ****P <0.0001, ns: not significant.

**Figure S10. Validation the effect of mitigated steatotic donor liver LT IRI by pyruvate acid from *Lachn.* in rats with liver cirrhosis. (A,B)** Western blot analysis of inflammatory markers in the liver of LC+ABX rats post-intervention with Pyruvic, Valeric, and Butyrate acids, following transplantation of ND-D and HFD-D livers. **(C,D)** qPCR (C) and ELISA (D) assessments of immune-related inflammatory markers in the liver and portal vein serum of LC+ABX rats intervened with Pyruvic, Valeric, and Butyrate acids, post-transplantation. **(E)** Immunofluorescence semi-quantitative analysis of macrophage infiltration in the transplant liver of LC+ABX rats post-intervention with Pyruvic, Valeric, and Butyrate acids, following transplantation of ND-D and HFD-D livers.

**Figure S11. Transcriptome sequencing analysis of liver transplantation after *Lachn.* intervention. (A)** Volcano plot of differential genes in liver transcriptomes post-transplant from normal and steatotic donors to various rat model including NC-R, LC-R, LC+Lachn.-R, LC+ABX-R, and LC+ABX+Lachn.-R rats. **(B)** Volcano plot of differential metabolites in liver transcriptomes post-transplant from normal and steatotic donors to the various rat models.

**Figure S12. Pyruvate acid mitigates** [**ferroptosis**](file:///D:\2\æéè¯å¸\Dict\7.5.2.0\resultui\dict\?keyword=ferroptosis) **in transplanted liver IRI by inhibiting ALOX15 expression. (A)** Heatmap of differential genes post-transplant from normal and steatotic donors to NC-R, LC-R, LC+Lachn.-R, LC+ABX-R, and LC+ABX+Lachn.-R rats. **(B)** Analysis of Alox15 expression levels correlation with pyruvate in transplanted livers. **(C-E)** Tissue immunofluorescence for ferroptosis markers in livers post-transplant from normal donors to the various rat models (Scale bar = 50 μm).

**Figure S13. Pyruvate acid mitigates** [**ferroptosis**](file:///D:\2\æéè¯å¸\Dict\7.5.2.0\resultui\dict\?keyword=ferroptosis) **and IRI in transplanted liver IRI by inhibiting ALOX15 expression. (A,B)** Oil Red O staining in HepG2 and RPH (Scale bar = 50 μm). **(C,D)** Expression of ALOX15 and ferroptosis markers in normal and steatotic HepG2 cells under IRI model after pyruvate intervention. **(E,F)** Investigation of Alox15 overexpression, knockdown, and pyruvate intervention in HepG2 cell lines and assessing inflammatory and ferroptosis markers expression under IRI model. **(G)** Alox15-Foxo3-pyruvate correlation analysis.

**Figure S14. Inhibition of ALOX15 significantly reduces ferroptosis in both normal and steatotic donor liver IRI. (A,B)** H&E and TUNEL assays to assess the severity of IRI and semi-quantitative analysis in transplanted livers from normal and steatotic donors to LC-R, LC+ML351-R, LC+ABX-R, and LC+ABX+ML351-R rats (Scale bar = 20μm). **(C,D)** Measurement of ALT, AST, and inflammatory marker levels in in transplanted livers from normal and steatotic donors to LC-R, LC+ML351-R, LC+ABX-R, and LC+ABX+ML351-R rats. **(E)** Tissue immunofluorescence to assess neutrophil and macrophage infiltration levels and perform semi-quantitative analysis in livers post-transplant from normal and steatotic donors to LC-R, LC+ML351-R, LC+ABX-R, and LC+ABX+ML351-R rats (Scale bar = 20 μm). **(F)** Tissue immunofluorescence for ferroptosis markers (GPX4, 4-HNE) expression in livers transplanted from normal and steatotic donors to LC-R, LC+ML351-R, LC+ABX-R, and LC+ABX+ML351-R rats (Scale bar = 20 μm). **(G,H)** Western blot analysis to evaluate changes in ferroptosis protein and inflammatory marker expression levels in livers from normal and steatotic donors post-transplant to LC-R, LC+ML351-R, LC+ABX-R, and LC+ABX+ML351-R rats (Scale bar = 20μm). Statistical significance was determined by unpaired Student t test. **P* <0.05, ***P* <0.01, ****P* <0.001, *****P* <0.0001, ns: not significant.

**Figure S15. Animal models validated inhibition of ALOX15 significantly reduces ferroptosis in both normal and steatotic donor liver IRI. (A,B)** H&E and TUNEL assays to evaluate the severity of IRI and perform semi-quantitative analysis in livers from normal and steatotic donors treated with AAV8-shAlox15, transplanted into rats with liver cirrhosis (LC-AAV8-shNC-R) and AAV8-shAlox15-treated liver cirrhosis (LC-AAV8-shAlox15-R) (Scale bar = 20μm). **(C,D)** Measurement of ALT, AST, and inflammatory markers in recipients of liver transplants from normal and steatotic donors treated with AAV8-shAlox15 into LC-AAV8-shNC and LC-AAV8-shAlox15 rats. **(E)** Tissue immunofluorescence to determine the levels and semi-quantitative analysis of neutrophil and macrophage infiltration in the livers post-transplant from normal and steatotic donors treated with AAV8-shAlox15 into LC-AAV8-shNC-R and LC-AAV8-shAlox15-R rats (Scale bar = 20 μm). **(F)** Tissue immunofluorescence for the expression of ferroptosis markers (GPX4, 4-HNE) in livers transplanted from normal and steatotic donors treated with AAV8-shAlox15 into LC-AAV8-shNC-R and LC-AAV8-shAlox15-R rats (Scale bar = 20 μm). **(G,H)** Western blot analysis to assess changes in the expression levels of ferroptosis proteins and inflammatory markers in livers from normal and steatotic donors treated with AAV8-shAlox15, transplanted into LC-AAV8-shNC-R and LC-AAV8-shAlox15-R rats. Statistical significance was determined by unpaired Student t test. **P* <0.05, ***P* <0.01, ****P* <0.001, *****P* <0.0001, ns: not significant.

**Figure S16. Elevated *Lachn.* abundance associated with alleviated liver IRI in LT recipients. (A)** Changes in ALT, AST, bilirubin, leukocytes, and neutrophil percentages within one week post-transplant in liver cirrhosis recipients with high and low *Lachn.* abundance receiving normal donor livers. **(B)** Analysis of the correlation between *Lachn.* abundance in feces and pyruvate levels in feces and portal vein serum of liver cirrhosis recipients. **(C)** Serum inflammatory marker levels post-transplant in liver cirrhosis recipients with high and low *Lachn.* abundance receiving normal and steatotic donor livers. **(D)** Tissue immunofluorescence analysis of neutrophil and macrophage infiltration in transplanted livers from normal and steatotic donors to recipients with different *Lachn.* abundance (Scale bar = 20 μm). **(E)** Tissue immunofluorescence for ferroptosis markers (GPX4, 4-HNE) expression levels in livers post-transplant from normal and steatotic donors to liver cirrhosis recipients with varying *Lachn.* abundance. **(F,G)** Analysis of perioperative complications and total hospital stay for liver transplant recipients with high and low *Lachn.* abundance. Statistical significance was determined by one-way and two-way ANOVA. **P* <0.05, ***P* <0.01, *****P*<0.0001, ns: not significant.
